# Nonprecious transition metal nitrides as efficient oxygen reduction electrocatalysts for alkaline fuel cells

**DOI:** 10.1126/sciadv.abj1584

**Published:** 2022-02-02

**Authors:** Rui Zeng, Yao Yang, Xinran Feng, Huiqi Li, Lauryn M. Gibbs, Francis J. DiSalvo, Héctor D. Abruña

**Affiliations:** Department of Chemistry and Chemical Biology, Cornell University, Ithaca, NY 14853, USA.

## Abstract

Hydrogen fuel cells have attracted growing attention for high-performance automotive power but are hindered by the scarcity of platinum (and other precious metals) used to catalyze the sluggish oxygen reduction reaction (ORR). We report on a family of nonprecious transition metal nitrides (TMNs) as ORR electrocatalysts in alkaline medium. The air-exposed nitrides spontaneously form a several-nanometer-thick oxide shell on the conductive nitride core, serving as a highly active catalyst architecture. The most active catalyst, carbon-supported cobalt nitride (Co_3_N/C), exhibited a half-wave potential of 0.862 V and achieved a record-high peak power density among reported nitride cathode catalysts of 700 mW cm^−2^ in alkaline membrane electrode assemblies. Operando x-ray absorption spectroscopy studies revealed that Co_3_N/C remains stable below 1.0 V but experiences irreversible oxidation at higher potentials. This work provides a comprehensive analysis of nonprecious TMNs as ORR electrocatalysts and will help inform future design of TMNs for alkaline fuel cells and other energy applications.

## Teaser

A systematic study explores transition metal nitrides for nonprecious oxygen reduction electrocatalysts in alkaline fuel cells.

## INTRODUCTION

Hydrogen fuel cells hold great promise for future automotive transportation because of their higher overall energy efficiency and potential zero carbon emissions when compared to internal combustion engines (*1*, *2*). However, the use of costly platinum (and other precious metals) for accelerating the sluggish oxygen reduction reaction (ORR) in proton exchange membrane fuel cells (PEMFCs) has precluded their widespread deployment for electric vehicle applications (*3*). Although extensive research efforts have been devoted to minimizing Pt usage and enhancing its intrinsic activity via alloying (*4*–*7*) and nanostructuring (*8*–*10*), replacing Pt with nonprecious metals or oxides represents a more promising strategy (*11*, *12*). Compared to PEMFCs in which the catalyst is estimated to contribute about 40% of the fuel cell stack cost (*13*), the primary advantage of anion exchange membrane fuel cells (AEMFCs) is that they enable the use of nonprecious transition metal–based ORR catalysts because of the improved catalyst stability in alkaline electrolytes (*14*). In an effort to facilitate the ORR kinetics in alkaline medium, a broad spectrum of nonprecious catalysts has been extensively studied, including metal-nitrogen-carbon (*15*, *16*), transition metal oxides (*13*, *17*–*19*), and perovskites (*20*, *21*). In particular, transition metal oxides, especially Co-Mn spinel oxides, have exhibited a power density of over 1 W cm^−2^ in membrane electrode assemblies (MEAs) (*22*). Unfortunately, the low intrinsic electrical conductivity of those semiconducting spinel oxides has prevented further improvements in their ORR activity (*23*). An ideal ORR electrocatalyst should have an active surface responsible for catalyzing the ORR process and a conductive bulk to facilitate the charge transfer. Thus, developing conductive nonprecious catalysts represents a viable and attractive approach to circumvent the conductivity challenges and enhance ORR performance.

Transition metal nitrides (TMNs) have emerged as attractive electrode materials because of their exceptional electrical conductivity, corrosion resistance, and mechanical robustness (*24*–*27*). Generally, TMNs are a family of interstitial alloys, formed by the incorporation of nitrogen atoms into the interstitial sites of the parent metals, with a combination of covalent, ionic, and metallic bonding characteristics (*24*, *25*). Owing to their unique electronic structures and remarkable electrical conductivity, TMNs have generated a great deal of research interests in electrocatalytic applications, involving hydrogen evolution/oxidation reaction (HER/HOR) (*28*, *29*), oxygen evolution/reduction reaction (OER/ORR) (*30*, *31*), and even nitrogen fixation reactions (*32*). In particular, the surfaces of TMNs have a tendency to be oxidized upon exposure to air or electrolyte, forming a thin layer of oxides/hydroxides that can serve as the active site for electrocatalytic reactions in alkaline environments (*33*, *34*). Consequently, the in situ formation of a nitride-core oxide-shell structure exhibits great advantages over its pure oxide counterpart. Zhang *et al.* (*33*) adopted a rapid N_2_ plasma treatment to synthesize metallic CoN nanowires, which formed a thin layer of active CoOOH on the surface before the OER process. The presence of a conductive CoN core could facilitate electron transport from the substrate to the catalyst surface, much better than its semiconducting Co_3_O_4_ counterpart. Hence, the CoN nanowires displayed improved OER activity with substantially reduced charge transfer resistance. Furthermore, Yu *et al.* (*34*) reported on a three-dimensional core-shell catalyst design consisting of NiFeN nanoparticles (NPs) uniformly distributed on NiMoN nanorods for alkaline seawater electrolysis. The outstanding performance arose from not only the in situ formed amorphous NiFe oxide/oxy(hydroxide) layer from the outer NiFeN NPs but also from the conductive interior NiMoN nanorods that ensured efficient charge transfer/transport. Thus, the natively formed active oxide/hydroxide shell, over the conductive nitride core, appears to satisfy the needs for an ideal electrocatalyst for the ORR in alkaline medium. Although some studies on TMNs as potential ORR catalysts in alkaline medium have been conducted (*30*, *35*–*37*), a comprehensive evaluation of TMNs as alkaline ORR electrocatalysts remains unfulfilled, primarily as a result of different synthetic strategies being adapted by different research groups. Moreover, most electrocatalytic performance evaluations of TMNs have been largely based on rotating disk electrode (RDE) experiments. Few research investigations have used TMNs as cathode catalysts for MEA testing in AEMFCs (*38*, *39*).

Here, we report on a group of nonprecious TMNs as potential ORR catalysts in alkaline medium. We first synthesized a family of carbon-supported metal nitrides (M*_x_*N/C, M = Ti, V, Cr, Mn, Fe, Co, Ni, *x* = 1 or 3) via a facile nitridation strategy and systematically studied their morphologies and structures. Using surface and elemental characterization tools, we established that all the synthesized nitrides adopt a nitride-core and oxide-shell configuration upon exposure to the atmosphere. Electrochemical measurements in alkaline electrolyte showed that Co_3_N/C, MnN/C, and Fe_3_N/C displayed promising ORR activity, with Co_3_N/C exhibiting the highest ORR performance, comparable to commercial Pt/C. We then demonstrate that a peak power density (PPD) of 700 mW cm^−2^ can be achieved with Co_3_N/C as cathode catalyst in an AEMFC, representing the highest MEA performance, among reported nitride cathode catalysts. Operando x-ray absorption spectroscopy (XAS) suggested that while Co_3_N/C remains stable at potentials below 1.0 V, versus the reversible hydrogen electrode (RHE), it undergoes marked oxidation at more positive potentials. This work may offer some insights into the design and development of active and durable TMNs as electrocatalysts for alkaline fuel cells and other energy systems and technologies.

## RESULTS AND DISCUSSION

### Structural and surface characterization of catalysts

**Fig. 1.**
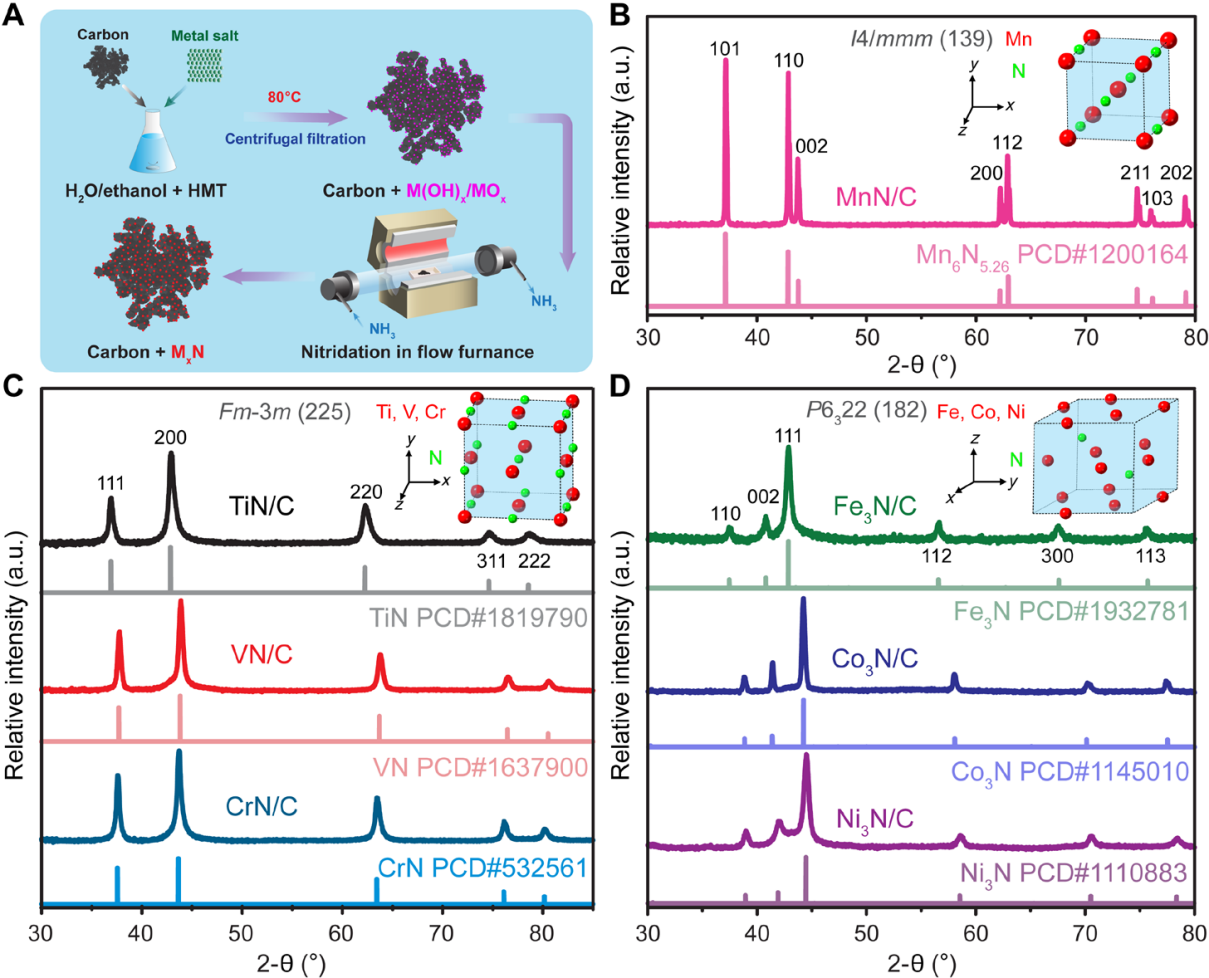
Synthesis and structural characterization of carbon-supported 3d metal nitrides. (**A**) Schematic synthesis procedure of metal nitrides (M*_x_*N) supported on high–surface area carbon via nitridation with ammonia. HMT, hexamethylenetetramine. (**B** to **D**) XRD patterns of as-synthesized M*_x_*N/C compared with patterns from Pearson’s Crystal Data (PCD) database and their atomic arrangement models with different structures. MnN/C, TiN/C, VN/C, CrN/C, Fe_3_N/C, Co_3_N/C, and Ni_3_N/C were prepared at 800°, 800°, 600°, 700°, 400°, 360°, and 300°C, respectively. a.u., arbitrary units.

A family of carbon-supported TMNs was prepared via a nitridation reaction with ammonia, as shown in Fig. 1A. The introduction of a carbon support promotes dispersion of TMNs and hence maximizes nitride utilization. The crystal structures of the as-synthesized M*_x_*N/C were first investigated with powder x-ray diffraction (XRD). The XRD patterns of TiN/C, VN/C, and CrN/C displayed the same characteristic spectral patterns, typical of the NaCl type face-centered cubic (fcc) structure (Fig. 1B and fig. S1, A to C), in which the metal atoms adopt an fcc arrangement, and the nitrogen atoms are located at the octahedral sites with a stoichiometric ratio of 1:1, as can be visualized from the crystal model (Fig. 1B). In contrast, manganese nitride is known to form multiple phases with different nitrogen contents (*40*–*42*). Among them, θ-MnN represents the most nitrogen-rich phase with an ideal Mn:N composition ratio of 1:1. However, some reports refer to it as Mn_6_N_5_ or Mn_6_N_5+*x*_ (*40*, *41*), indicating a random distribution of some nitrogen vacancies in the crystal lattice (*40*). The as-synthesized MnN/C exhibited XRD patterns characteristic of NaCl-type face-centered tetragonal (fct) structure, corresponding to θ-MnN, with a noticeable distortion in the ***c*** direction (Fig. 1C and fig. S1D). Meanwhile, the XRD spectral features of Fe_3_N/C, Co_3_N/C, and Ni_3_N/C revealed that they share the same hexagonal crystal structure with six metal atoms and two nitrogen atoms per unit cell (Fig. 1D and fig. S1, E and F), exhibiting a lower nitrogen content when compared to early TMNs. The complete conversion of the metal oxide/hydroxides to nitrides by ammonia treatment was further confirmed by microscopic structural characterization with high-resolution transmission electron microscopy (HRTEM) (fig. S3), in which the lattice spacings of nitrides were consistent with those of their corresponding standard nitride references. The particle size distributions of the metal nitride NPs were analyzed with scanning transmission electron microscopy (STEM) images (fig. S2). The average particle sizes were estimated to be around 15 to 20 nm for TiN/C, VN/C, CrN/C, Fe_3_N/C, and Ni_3_N/C but slightly larger for Co_3_N/C (~30 nm). In comparison, MnN/C displayed much larger particle size at around 200 nm since synthesis of small manganese nitride particles with high dispersion remains a challenge (*36*, *43*).

**Fig. 2.**
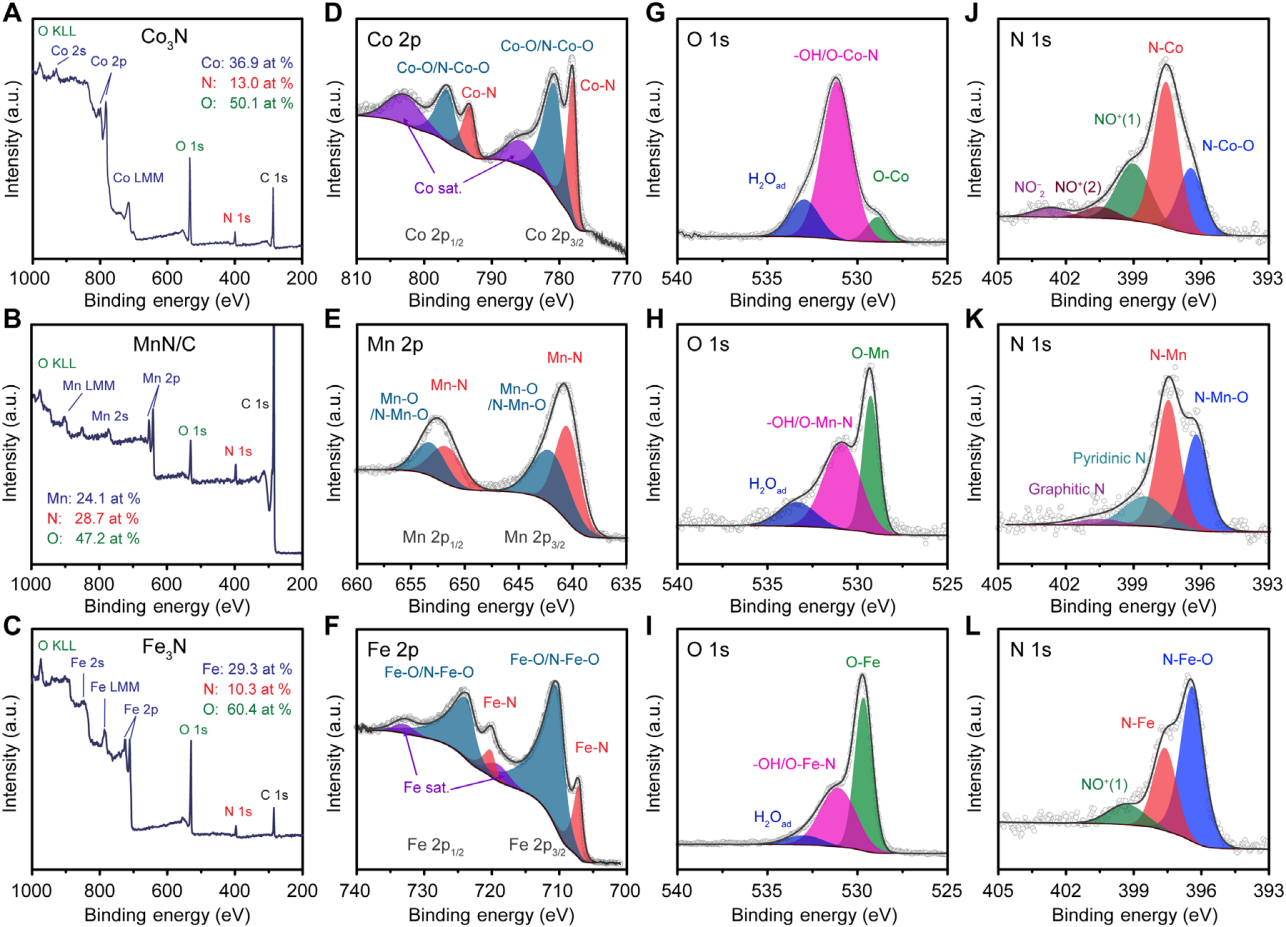
Surface environment investigation by XPS. Survey scan spectra (**A** to **C**) and deconvoluted high-resolution spectra of metal 2p orbitals (**D** to **F**), O 1s (**G** to **I**), and N 1s (**J** to **L**) from Co_3_N, MnN/C, and Fe_3_N. The atomic ratios of Co/Mn/Fe, N, and O were calculated on the basis of their 2p and 1s signals.

X-ray photoelectron spectroscopy (XPS) was used to probe the surface chemical environment of the metal nitrides as it provides surface-sensitive elemental and bonding information. To improve the signal-to-noise (S/N) ratio and minimize the possible contribution of nitrogen signals from the carbon support, metal nitrides were characterized without the use of a carbon support, except for manganese nitride. As shown in Fig. 2 (A to C) and fig. S4 (A to D), primary survey scans indicated that the surfaces of the metal nitrides are dominated by oxygen-containing species, likely due to natural oxidation when the sample is exposed to air (*23*, *42*). However, the compositional ratios between metal and nitrogen remained close to their bulk counterparts except for VN (fig. S4B) and MnN/C (Fig. 2B), suggesting that the surface oxidation process does not lead to a notable release of nitrogen species from the original nitride crystal lattice to the environment (*44*). Furthermore, high-resolution XPS spectra provided more bonding information for species identification, including nitride, oxynitride, and oxide/hydroxide species (Fig. 2, D to L, and fig. S4, E to O). As for the high-resolution scans of metal 2p orbitals (Fig. 2, D to F, and fig. S4, E to H), the spectra displayed characteristic spin-orbit splitting (2p_3/2_ versus 2p_1/2_). The components at low binding energies (low oxidation state) can be described as metal-nitrogen (M-N) bonding as they displayed metallic behavior (*45*). The emergence of spectral features at higher binding energies suggested the formation of oxides/hydroxides or oxynitrides arising from spontaneous surface oxidation. In addition to the primary peaks, distinct satellite features can also be observed at higher binding energies (Fig. 2, D and F, and fig. S4, E, F, and H), likely due to a combination of multiple electronic band or structural effects (*46*). Oxide formation was further evidenced from the sharp metal-oxygen (M-O) peak at lower binding energy in the O 1s spectra (Fig. 2, G to I, and fig. S4, F and I to K), indicative of the presence of O^2−^ ions in the form of metal oxides (*47*). In comparison, the broad peaks at ~531.0 eV are ascribed to hydroxide and oxynitride species, and the residual peaks at ~533.0 eV can be further assigned to adsorbed H_2_O (*46*, *48*). In contrast to MnN/C and Fe_3_N, Co_3_N showed a major O 1s peak at 531.1 eV in Fig. 2G, suggesting that the surface oxide species is dominated by hydroxide species rather than a pure oxide. In addition to the nitride phase and oxide surface, oxynitrides in the sample can be unambiguously identified from the peak splitting of the major N 1s peak. With the exception of CrN (*49*), all other nitrides displayed a similar 0.9- to 1.2-eV peak splitting (Fig. 2, J to L, and fig. S4, L to O), indicating the formation of oxynitrides. This ~1-eV shift of the oxynitride peak to lower binding energy, with respect to the nitride peak, has been reported to be due to increased charge transfer from metal to nitrogen (*50*). Consequently, the oxynitride phase represents an intermediate state connecting the nitride core and the oxide/hydroxide surface. In summary, the comprehensive XPS analysis revealed that all the metal nitride NPs exhibited a similar core-shell configuration with an oxide/hydroxide overlayer on the parent nitride core and likely with an interfacial buffer layer of oxynitride between them.

**Fig. 3.**
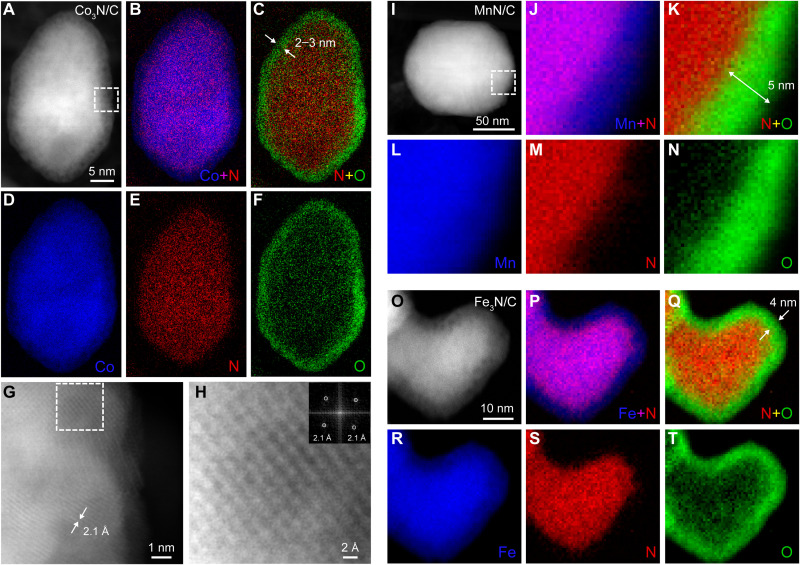
Elemental composition and distribution of Co_3_N/C, MnN/C, and Fe_3_N/C catalysts by STEM-EELS. (**A**) High-angle annular dark-field (HAADF)-STEM image of an elliptical Co_3_N particle. (**B** to **F**) Corresponding EELS elemental mapping of Co (D), N (E), O (F), and their composite mapping of Co + N (B) and N + O (C). (**G**) Atomic-scale HADDF-STEM image acquired from the dashed box in (A). (**H**) Atomic-scale HADDF-STEM image zoomed in from the dashed box in (G). The inset shows the corresponding fast Fourier transform image. (**I**) HADDF-STEM image of a MnN particle. (**J** to **N**) EELS elemental mapping of Mn (L), N (M), O (N), and their composite mapping of Mn + N (J) and N + O (K) collected from the dashed box in (I). (**O**) HADDF-STEM image of a corresponding Fe_3_N particle. (**P** to **T**) Corresponding EELS elemental mapping of Fe (R), N (S), O (T), and their composite mapping of Fe + N (P) and N + O (Q).

The atomic-scale microstructures and chemical compositions of Co_3_N/C, MnN/C, and Fe_3_N/C were further investigated by aberration-corrected high-angle annular dark-field STEM along with electron energy loss spectroscopy (EELS) elemental mapping. The atomic-scale STEM images exhibited d-spacings of 2.1 Å on the edge near the surface of the Co_3_N NPs (Fig. 3, G and H, and fig. S6, A to D), corresponding to the CoO{200} plane. The potential existence of CoO within the surface oxide layer is nominally consistent with the previous XPS analysis. The elemental distributions of Co, N, and O, derived from EELS analysis with an energy resolution of 1.5 eV (fig. S5), provided more compelling evidence for the local chemical environment. The as-synthesized Co_3_N/C catalyst exhibited a homogeneous distribution of Co and N (Fig. 3, B, D, and E, and fig. S7), confirming the dominant presence of the nitride phase. In contrast, the O content concentrated near the surface of the particle, giving rise to a hollow elliptical oxide ring with a thickness of 2 to 3 nm (six to nine atomic layers), as revealed from the O mapping and the composite mapping of N and O (Fig. 3, C and F, and fig. S7). These observations indicate that a layer of oxide naturally forms on the freshly prepared Co_3_N once exposed to air, consistent with the XPS analysis of Co_3_N and similar to our previous work (*23*). Furthermore, EELS analysis of Co_3_N/C after long-term exposure to air showed no further increase of the oxidation layer (fig. S8), indicative of good air chemical stability of the nitride-core oxide-shell architecture in the atmosphere. This finding suggested that this native oxide layer might act as a passivation layer to inhibit further oxidation, as is well known for aluminum (*51*).

Similar core-shell structures were also confirmed through STEM-EELS analysis of MnN/C and Fe_3_N/C catalysts. The elemental mapping of a MnN particle near the surface can be divided into two regions: a region with a uniform distribution of Mn and N in the core surrounded by an O-rich region (Fig. 3, J, L, and M, and figs. S9 and S10). Compared with the EELS features in the nitride-core region (fig. S9H), the shell displayed a strong O K-edge without a detectable N signal, again suggesting O enrichment of the surface layer (fig. S9G). The composite maps of N and O revealed an oxide surface region with a thickness of 5 nm (Fig. 3K and figs. S9C and S10C), slightly larger than that of Co_3_N/C. The d-spacings of 2.2 Å correspond to MnO{200} in fig. S10H. Similarly, the EELS maps of Fe, N, and O in Fe_3_N/C also displayed a core-shell structure, with O concentrated near the surface (Fig. 3, O to T). The surface oxide layer was measured to be 3 nm, close to that of Co_3_N/C. EELS maps in figs. S11 and S12 further confirmed this observation. The atomic-scale STEM image near the particle surface revealed a lattice spacing of 2.5 Å, which likely originates from either FeO{200} or Fe_3_O_4_{311} (fig. S12H), in agreement with the EELS mapping analysis and XPS results.

### Electrocatalytic characterization of catalysts

**Fig. 4.**
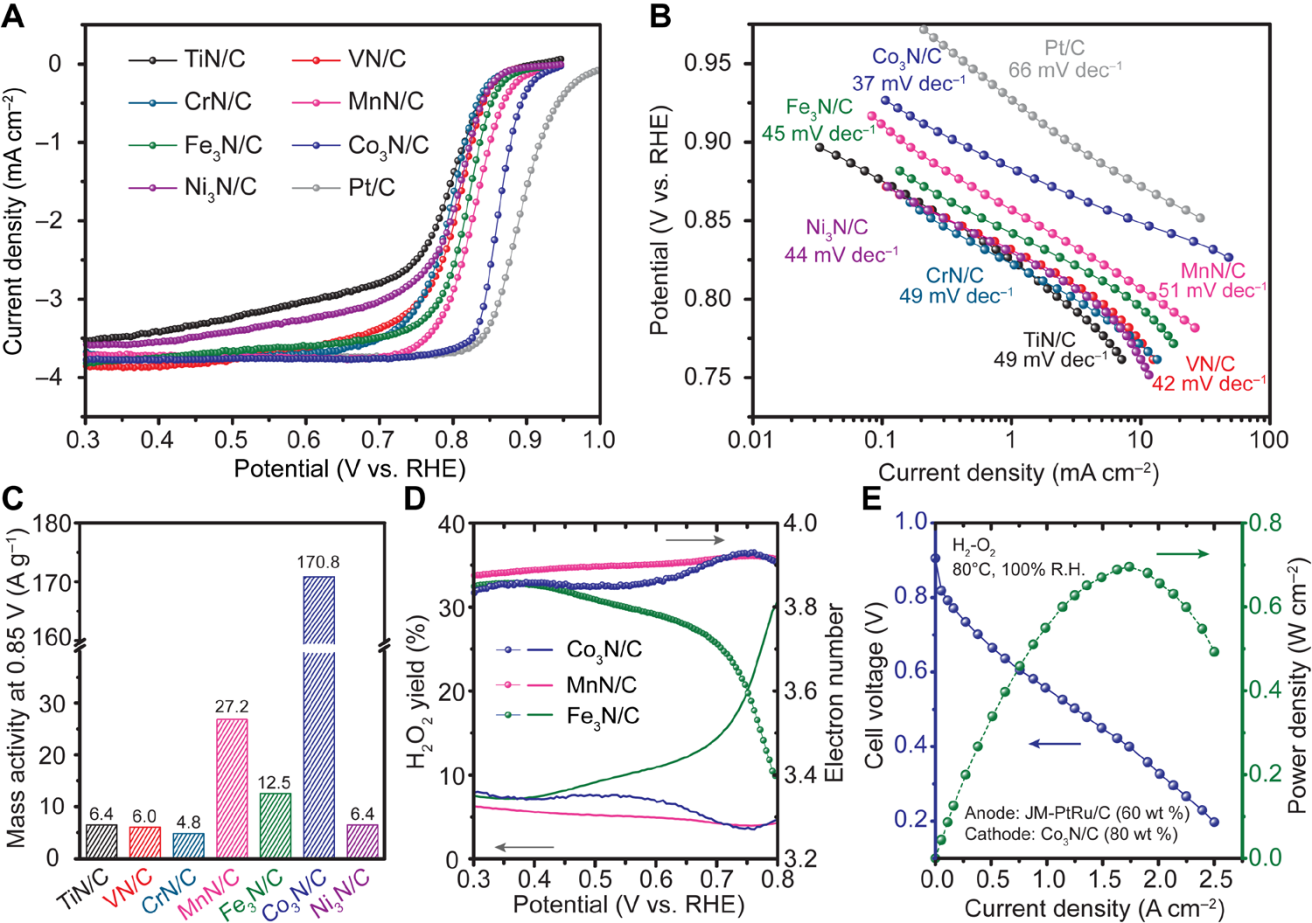
Electrochemical evaluation of M*_x_*N/C as ORR catalysts in alkaline medium. (**A**) RDE polarization curves of M*_x_*N/C [20 weight % (wt %), 50 μg_nitride_ cm^−2^] and commercial Pt/C (20 wt %, 25 μg_Pt_ cm^−2^) in O_2_-saturated 1 M KOH. The scan rate was 5 mV/s, and the rotation rate was 1600 rpm. The current was normalized to the geometric area of the electrode. (**B**) ORR Tafel plots were extracted from (A), and the kinetic current density was determined from the Koutecký-Levich equation. (**C**) Comparison of the MA of all M*_x_*N/C measured at 0.85 V versus RHE. (**D**) H_2_O_2_ yield (bottom region) and electron transfer number (top region) of Co_3_N/C, MnN/C, and Fe_3_N/C, measured with RRDE. (**E**) AEMFC performance with cathode catalyst of 80 wt % Co_3_N/C (1.8 mg_Co3N_ cm^−2^) and anode catalyst of 60 wt % commercial PtRu/C (0.4 mg_PtRu_ cm^−2^). The cell was operated at 80°C with fully humidified H_2_ [500 standard cubic centimeters per minute (sccm)] and O_2_ (500 sccm) with a gas back pressure of 0.2 MPa.

The ORR activities of the M*_x_*N/C catalysts were evaluated in O_2_-saturated 1 M KOH with an RDE. As shown in the ORR polarization curves in Fig. 4A and fig. S13, Co_3_N/C, MnN/C, Fe_3_N/C, VN/C, and CrN/C displayed a well-defined mass transport–limited current density (*j*_lim_) approaching 3.7 mA cm^−2^ at 1600 rpm, suggesting a dominant four-electron reduction process. It should be noted that the *j*_lim_ reached 3.7 mA cm^−2^, rather than the commonly reported value of 5.5 mA cm^−2^ in 0.1 M KOH (*52*, *53*), since the oxygen solubility in 1 M KOH is ~70% of that in 0.1 M KOH (*54*). On the other hand, TiN/C and Ni_3_N/C exhibited notably lower diffusion-limited current densities, indicating a high H_2_O_2_ yield via the two-electron reduction process. Based on the half-wave potential (E_1/2_) and mass activity (MA) measured at 0.85 V versus RHE in Fig. 4C and fig. S14A, Co_3_N/C, MnN/C, and Fe_3_N/C outperformed the other four nitride catalysts, with Co_3_N/C exhibiting the highest MA at ~170 A g^−1^ and an E_1/2_ of 0.862 V versus RHE, a value that is within 30 mV of that of benchmark Pt/C. Overall, the ORR activity of metal nitride electrocatalysts evaluated by RDE decreased in the following sequence: Co_3_N/C > MnN/C > Fe_3_N/C > VN/C ≈ Ni_3_N/C > CrN/C > TiN/C (table S1). These trends are consistent with previous ORR results with early TMNs (*35*) and spinel oxides (*13*), suggesting that the active sites for the ORR processes originate from the formed oxy/hydroxide surface in solution (*34*). While the metal nitride catalysts displayed ORR activity lower than that of Pt/C, all of them exhibited substantially lower Tafel slopes with values below 51 mV dec^−1^, with Co_3_N/C reaching an exceptionally low value of 37 mV dec^−1^ (Fig. 4B). While some works have attributed the diminished Tafel slopes to enhanced reaction kinetics (*55*), the less active metal oxide catalysts usually showed smaller Tafel slopes in alkaline medium when compared to Pt group metal catalysts (*56*). Thus, the Tafel slope should not necessarily be used as an activity descriptor but rather as a possible indicator of the rate-determining step (RDS). The Tafel slope of Pt/C (66 mV dec^−1^) is consistent with previous work (*13*, *56*), indicating that the ORR kinetics on Pt/C in alkaline medium are controlled by the first proton transfer process (*56*). In contrast, the Tafel slope decreases to 40 mV dec^−1^ when the second electron transfer process becomes the RDS (*56*), suggesting that the ORR kinetics on Co_3_N/C and VN/C are likely limited by this process. For other nitride catalysts, their ORR reaction rates are determined by both processes. Thus, the Tafel slope analysis can provide some insights into the ORR reaction mechanism. The ORR product selectivity of the three best catalysts Co_3_N/C, MnN/C, and Fe_3_N/C was assessed by the rotating ring disk electrode (RRDE) technique, in which a Pt ring held at a constant potential (1.3 V versus RHE) is used to capture the electrogenerated H_2_O_2_ from the disk electrode (Fig. 4D and fig. S14, C to E). Specifically, MnN/C and Co_3_N/C exhibited H_2_O_2_ yields of ~5% and less than 8%, respectively, approaching that of Pt/C (3 to 4%; fig. S14F). Fe_3_N/C, which displayed a substantial generation of H_2_O_2_ at 0.8 V, had the H_2_O_2_ yield decreased to ~12% at 0.6 V and ~8% at 0.3 V, which is likely due to the role of Fe in catalyzing peroxide formation as in the Fenton reaction.

To evaluate the ORR capabilities of the most active nitride catalyst under real fuel cell working conditions, Co_3_N/C at a mass fraction of 80% was used as the ORR cathode catalyst in a MEA in an H_2_-O_2_ fuel cell test, with 60% PtRu/C as the anode and a quaternary ammonium poly(*N*-methyl-piperidine-co-p-terphenyl) (QAPPT) as the anion exchange membrane (*22*). As presented in Fig. 4E, the Co_3_N/C cathode achieved an open circuit voltage of 0.91 V and a PPD of 700 mW cm^−2^ at 1.75 A cm^−2^, representing the highest reported PPD in the literature based on nitride cathode catalysts for alkaline fuel cells (*38*, *39*). This remarkable performance validated the RDE results and we believe arises from the improved conductivity of Co_3_N/C, compared to its oxide counterpart, and facile mass transport due to the thin catalyst layer at a high mass fraction of 80 weight % (wt %) (*22*).

Aiming to develop practical nonprecious metal cathodes for AEMFCs, TMN electrocatalysts need to not only demonstrate high initial ORR activity but also achieve long-term durability. The stability of Co_3_N/C was investigated following an accelerated durability test (ADT) protocol recommended by the U.S. Department of Energy: potential cycling between 0.60 and 0.95 V versus RHE for 10,000 cycles in O_2_-saturated 1 M KOH. Co_3_N/C exhibited remarkable durability with an E_1/2_ decay of only 14 mV, comparable to that of Pt/C (17 mV; fig. S16, A and B). The activity decay of Co_3_N/C was ascribed to the loss of surface area and degradation of active materials during the potential cycles. The dissolution analysis showed that some cobalt dissolved into the electrolyte during the ADT test (table S2), suggesting that particle dissolution could contribute to the loss of surface area. Furthermore, the original cobalt nitride species likely degraded into an oxide/hydroxide, which could be inferred from the oxide peak formation at 0.95 V, by comparing the cyclic voltammetric (CV) profiles before and after potential cycles (fig. S17). Detailed XPS spectra and STEM-EELS images further revealed that Co_3_N NPs were converted into rod-shaped Co_3_O_4_ with negligible nitrogen content (figs. S18 to S20), which could account for the activity decay. These observations are consistent with results obtained from realistic fuel cell stability testing in MEAs, in which Co_3_N/C cathodes degraded to cobalt oxyhydroxides from the initial nitride phase (fig. S21, A and B). Consequently, the durability testing pointed toward the importance of further improving the long-term stability/durability of nitride-based cathode electrocatalysts. Co_3_N/C was tested at oxidizing potentials beyond 1.0 V to investigate its stability at high potentials and the possibility of catalyzing the OER. In CV profiles, Co_3_N/C exhibited an oxidation peak at ~1.0 V when the applied potential increased to 1.2 V and the oxidation peak continued to grow when applied potentials increased further to 1.4 and 1.6 V (fig. S22A). The ORR polarization profiles of Co_3_N/C after CV cycles to 1.2 to 1.6 V showed a substantially lower ORR activity (fig. 22B), relative to that with CV cycles to 1.0 V, suggesting a “threshold potential” of ~1.0 V for Co_3_N/C to maintain its activity and structural stability. This finding suggests an underlying reaction mechanism involving the formation of different species at potentials above 1.0 V.

### Operando XAS of Co_3_N/C

**Fig. 5.**
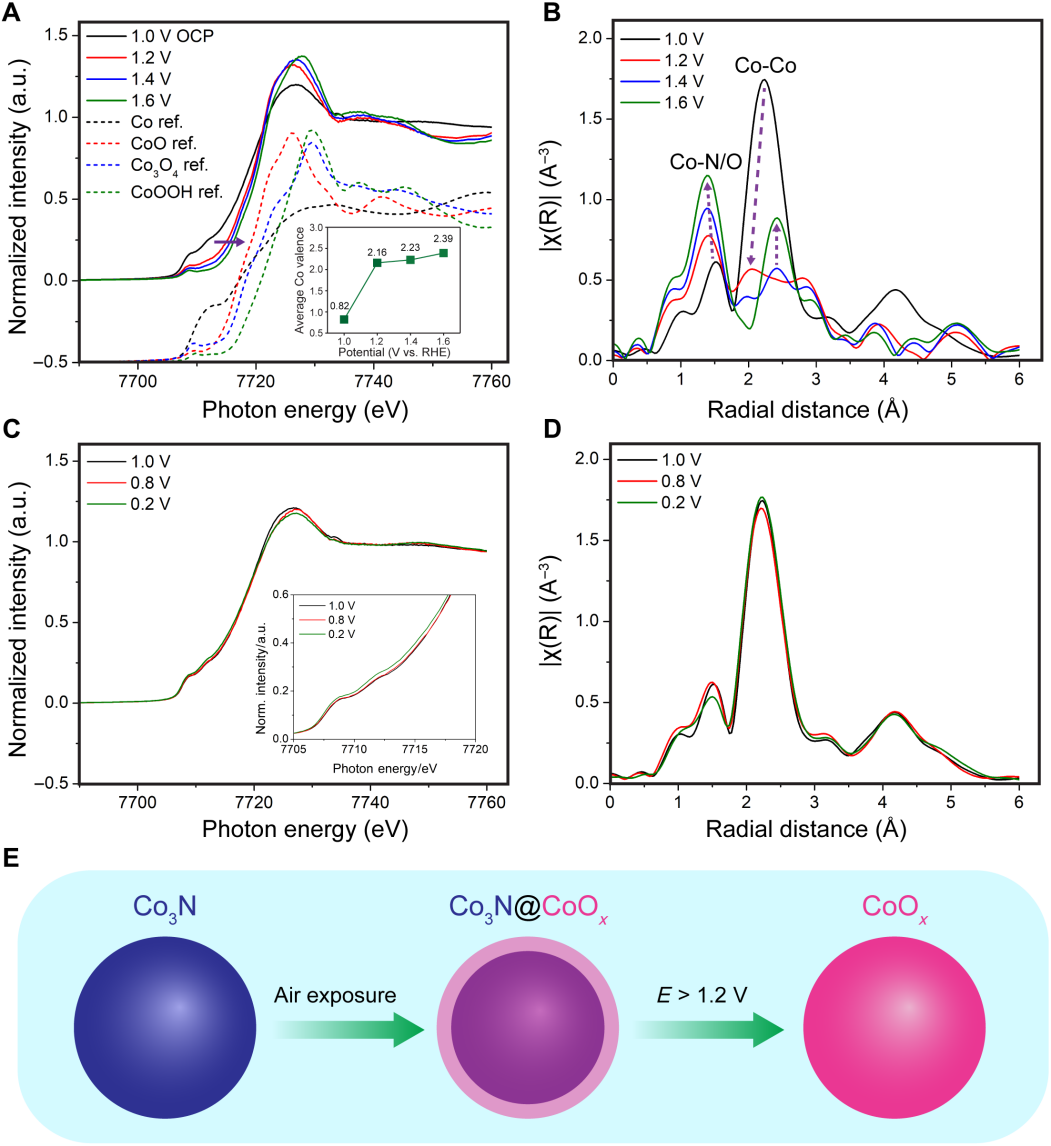
Operando XAS studies of the stability of Co_3_N/C under electrochemical conditions. (**A**) Operando XANES spectra of Co_3_N/C under steady state at 1.0 V (OCP) followed by applied oxidative potentials from 1.2 to 1.6 V versus RHE, and comparison with XANES reference spectra (a Y-offset of 0.5 was applied to better show the differences). The inset shows the average Co valences at 1.2 to 1.6 V based on an LCF analysis. (**B**) Operando EXAFS spectra of Co_3_N/C at 1.0 to 1.6 V with *k*^3^-weighting Hanning window and no phase correction, showing the marked changes in the Co-N/O and Co-Co bonds at different applied oxidative potentials. (**C** and **D**) Operando XANES and EXAFS spectra of Co_3_N/C at reducing potentials from 1.0 V down to 0.2 V. (**E**) Schematic illustration of Co_3_N particle undergoing surface oxidation upon air exposure and complete oxidation after applying oxidative potentials above 1.2 V.

Operando synchrotron-based XAS was used to understand the activity and structural stability of Co_3_N/C under real-time electrochemical conditions in alkaline medium. The cell design and experimental details can be found in Materials and Methods. The electrochemical response of Co_3_N/C in a homemade XAS cell was comparable (fig. S23) to that in a conventional electrochemical cell (fig. S13L). Constant potentials from 0.2 to 1.6 V versus RHE were applied, while operando XAS spectra were acquired, to investigate the Co_3_N/C, under steady state in both reduction and oxidation conditions in alkaline medium. Operando x-ray absorption near-edge structure (XANES) spectra of the Co K-edge were acquired and calibrated against a Co metal foil with an edge energy of 7709.0 eV (Fig. 5A). The XANES spectrum of the as-synthesized Co_3_N/C at 1.0 V [open circuit potential (OCP)] showed an intense absorption peak at around 7726 eV, corresponding to the transition from core-level 1s to empty 4p orbitals. The white line of Co_3_N/C at 1.0 V is more similar to the sharp feature of CoO (red dashed line) rather than the plateau of metallic Co (dashed black line) at around 7726 eV. However, at 1.0 V, Co_3_N/C exhibited a broad pre-edge at around 7709 eV, which resembled the metallic Co features, more than Co oxides. When the potential was increased from 1.0 to 1.2 V versus RHE, the Co K-edge showed a notable shift to higher energy along with a higher intensity of the absorption peak, and the broad pre-edge changed to a sharper feature like those in cobalt oxide references. This change suggests an increase in the Co valence state and formation of Co oxides from the Co_3_N/C at 1.2 V. Further increases in the potential to 1.4 and 1.6 V caused a progressive positive shift of the edge energy, as indicated by the arrow in Fig. 5A, suggesting further oxidation of Co at higher applied potentials.

To investigate the change of the Co valence more quantitatively, we performed linear combination fitting (LCF) analysis using metallic Co(0), CoO(II), Co_3_O_4_(II,III), and CoOOH(III) as XANES spectral references (fig. S24, A and B, and table S3). As summarized in the inset of Fig. 5A, the data exhibit a trend in which the calculated average Co valence increases at higher applied potentials. The LCF analysis showed that Co_3_N/C at 1.0 V has an average valence of 0.82 ± 0.04 based on metallic Co and CoO references (fig. S24A). When the potential was increased to 1.2 V, the average valence increased markedly to 2.16 ± 0.02 based on the Co oxide references (fig. S24B). Further increases in potentials to 1.4 and 1.6 V led to average Co valences of 2.23 and 2.39, respectively (fig. S24, C and D). The noticeable mismatch between the XANES spectrum at 1.0 V and the LCF fitting results is likely due to the oxide shell and the large differences in crystal structures among Co_3_N (hexagonal), Co, and CoO (both are cubic). A metallic Co reference was used here since no XANES reference of a cobalt nitride is available. In addition, the thin cobalt oxide layer naturally formed on the surface of Co_3_N may also affect the average Co valence. The LCF fits well the experimental XANES spectra at 1.2 to 1.6 V, as indicated by the smaller fitting errors, reduced χ^2^ values (table S3).

Besides the valence information from XANES analysis, the operando extended x-ray absorption fine structure (EXAFS) spectra provided valuable insights on the coordination environment (*57*) of Co under reaction conditions (Fig. 5B). Fourier-transformed EXAFS spectra were processed with a *k*^3^-weighting Hanning window (3 to 12 Å) and without phase correction. EXAFS spectra of Co_3_N/C at 1.0 V (OCP) showed the characteristic Co-N bond at an *R* value of 1.5 Å, a pronounced Co-Co bond at 2.2 Å (nearest neighbors), and an additional peak at 4.2 Å (further coordination shells). It should be noted that the values of the radial distances (*R*) are smaller than the actual bond lengths by ~0.2 to 0.5 Å due to phase shifts from both the central and scattering atoms. When the potential was increased to 1.2 V, the amplitude of the EXAFS signal, |χ(*R*)| decreased markedly from 1.8 to less than 0.6 Å^−3^. This indicates that the structure of Co_3_N/C has been markedly perturbed under oxidation at 1.2 V becoming more disordered with a larger EXAFS Debye-Waller factor, σ^2^, based on the EXAFS equation (eq. S1). Further oxidation at 1.4 and 1.6 V showed a feature emerging at 4.4 Å, with an increasing magnitude to 0.6 and 0.9 Å^−3^, respectively. This suggests the formation of more ordered cobalt oxides at very high oxidation potentials. The 4.4-Å Co-Co peak at 1.4 and 1.6 V is 0.2 Å longer than that of Co_3_N/C, consistent with the theoretical increase in the bond lengths (0.24 Å) from Co_3_N to CoOOH or Co_3_O_4_. Changes in Co-N and Co-O are discussed together as “Co-N/O” since the differences between Co-N and Co-O bond lengths are too small to be differentiated by EXAFS, given its limited spatial resolution (Fig. 5B). As the potential was increased from 1.0 to 1.2 V, the Co-N/O bond decreased slightly from 1.5 to 1.4 Å and remained at 1.4 Å at higher potentials of 1.4 and 1.6 V. This suggests that the destructive oxidation at 1.2 V caused not only a more disordered structure but also a N/O coordination shell closer to the central Co atom. Details of experimental radial distances and theoretical bond lengths and coordination numbers are summarized in tables S4 and S5.

In contrast to the marked changes at oxidation potentials above 1.0 V, operando XANES and EXAFS showed a much more stable behavior at lower potentials (Fig. 5, C and D). When the potential decreased from 1.0 V (OCP) to 0.8 V, both XANES and EXAFS showed little change. Only when the potential was decreased to the lower limit of 0.2 V was a slight negative shift of the edge energy in XANES observed, as indicated in the inset of Fig. 5C. In addition, a slightly lower magnitude of the Co-N/O peak at 1.5 Å was observed. Both changes indicate a slight partial reduction from Co_3_N to metallic Co. However, the changes under reducing potentials are much smaller than those at oxidizing potentials. It is likely that the thin surface oxide layer is reduced at low potentials, but the relative contents of the surface oxide layer may be too low to show up in the EXAFS signal. As shown in the schematic in Fig. 5E, the results unambiguously demonstrate that oxidizing potentials above 1.2 V will destroy the original structure of Co_3_N and generate Co oxide species, while reducing potentials, down to 0.2 V, have minimal impact on the structural integrity. Co_3_N/C has a safe “potential threshold” of 1.0 V but is not suitable to catalyze the OER at high oxidizing potentials in alkaline medium. However, numerous reports have used Co-based nitride materials for the OER in alkaline solution (*31*, *33*, *34*), which contradicts our operando XAS observations. The nitride catalysts exhibited a tendency toward complete oxidation with the collapse of the conductive-core-active-shell structure, leading to marked performance deterioration. Further treatment of the catalyst surface with a passivation layer could help mitigate the core from oxidizing under oxidative conditions and thus improve the durability and structural stability of TMNs for the ORR in alkaline medium (*58*). Moreover, this strategy may also benefit the long-term durability of TMNs as cathode catalysts for AEMFCs.

In summary, a class of carbon-supported TMN catalysts (M*_x_*N/C, M = Ti, V, Cr, Mn, Fe, Co, Ni, *x* = 1 or 3) for the alkaline ORR has been successfully prepared and systematically investigated. The bulk nitride crystal structures were thoroughly examined and verified by XRD and TEM, while XPS provided unambiguous evidence for the presence of a nitride-core-oxide-shell structure, which was further confirmed by extensive STEM-EELS analysis. Three candidates, Co_3_N/C, MnN/C, and Fe_3_N/C, displayed promising ORR activity, with the Co_3_N/C cathode catalyst reaching an impressive PPD of 700 mW cm^−2^ in hydrogen-oxygen fuel cell testing. We also used operando XAS to investigate the stability of Co_3_N/C under both oxidative conditions (1.0 to 1.6 V versus RHE) and reducing potentials (1.0 to 0.2 V versus RHE). The results suggest that Co_3_N/C remains stable at potentials below 1.0 V but underwent severe degradation at potentials beyond 1.2 V, and hence, a passivation layer coating may be needed to maintain the structural stability and ensure long-term durability under OER conditions. These findings pave the way to the design of TMN-based electrocatalysts for a variety of renewable energy applications.

## MATERIALS AND METHODS

### Chemicals and materials

Vanadium(III) chloride (VCl_3_, ≥99.0%), chromium(III) chloride hexahydrate (CrCl_3_·6H_2_O, ≥98.0%), manganese(II) acetate tetrahydrate [(CH_3_COO)_2_Mn·4H_2_O, ≥99.0%], iron(II) acetate [Fe(CO_2_CH_3_)_2_, ≥99.99%], nickel(II) acetate tetrahydrate [Ni(OCOCH_3_)_2_·4H_2_O, ≥99.0%], hexamethylenetetramine (HMT; C_6_H_12_N_4_, ≥99.0%), and potassium hydroxide (KOH, 99.99%) were purchased from Sigma-Aldrich. Titanium(IV) oxide (TiO_2_, ≥99.0%) was purchased from EMD Chemicals. Cobalt(II) chloride hexahydrate (CoCl_2_·6H_2_O, 99.9%) was purchased from Alfa Aesar. HiSPEC Platinum 20% on carbon (20 wt % Pt/C) with an average particle size of 3 nm was from Johnson Matthey Fuel Cells. High–surface area Ketjen Black carbon powder (EC-600JD) was purchased from AkzoNobel. Absolute ethanol, used during synthesis, was purchased from Fisher Scientific. High–surface area Ketjen Black EC-300J supported PtRu catalysts (PtRu/C, 60 wt %), D2021 Nafion dispersion (20 wt %), and AvCarb MGL190 carbon paper (190 μm thick) were purchased from the Fuel Cell Store. QAPPT (ion-exchange capacity = 2.50 ± 0.05 mmol/g, 25 ± 2 μm thick) membrane and ionomer binder were purchased from Eve Energy. Deionized water (18.2 megohm·cm) was obtained from a Barnstead Nanopure water purification system. All chemicals were used as received, without further purification.

### Synthesis of metal nitride catalysts

A series of 3d metal nitrides was prepared by nitridation of metal oxide/hydroxide loaded on carbon as described in (*19*). In a typical synthesis, 0.4 mmol of metal salt and 224 mg of HMT were first dissolved in 30 ml of H_2_O/ethanol mixture (vol. 1:1) in a 40-ml Erlenmeyer flask. For a targeted nitride loading (20 wt %), the corresponding amount of high–surface area Ketjen Black carbon powder was transferred into the solution and ultrasonicated for 30 min. The flask was sealed, heated to 80°C under vigorous magnet stirring (1300 rpm) in the oil bath, and allowed to react for 1.5 hours. After the suspension was cooled down to room temperature, the precursor was separated from the residual solution via centrifugation at a rotation rate of 7000 rpm and washed with an H_2_O/ethanol mixture four times. The resulting precipitate was dried at 60°C in an oven for 6 hours and ground into a powder in an agate mortar. A small portion of the powder was transferred to an alumina ceramic boat and annealed in a flow furnace with flowing NH_3_ gas at 100 ml min^−1^. The temperature ramping rate and annealing time were controlled to 8°C min^−1^ and 2 hours, respectively. After the nitridation process, the samples were cooled to room temperature in NH_3_ and ground into powders. For the preparation of TiN/C, TiO_2_ and Ketjen Black carbon power were mixed and ground for 30 min before nitridation in a flow furnace. For the preparation of MnN/C, the precursor was obtained by drying the metal salt and carbon powder mixture solution under magnetic stirring at room temperature with purged N_2_. Instead of directly mixing the metal salt with HMT and carbon powder in solution, a syringe pump was used to minimize the particle size during the precursor synthesis for Co_3_N/C. The metal salt was first dissolved in a 10-ml H_2_O/ethanol mixture and then pumped into the heated flask containing HMT and carbon powder solution at a pumping rate of 0.111 ml min^−1^. Other procedures remained the same.

### Material characterizations

A Rigaku Ultima IV diffractometer operated at 1.76 kW (40 kV and 44 mA) was used to examine the crystal structures of the as-synthesized metal nitrides. The powder XRD patterns were collected from 30° to 80° at a scan rate of 4° min^−1^ and step size of 0.02°. The XRD background was subtracted by the Rigaku PDXL software. The morphologies and structures of the as-prepared samples were examined with a FEI Tecnai 12 BioTwin TEM (120 kV) and the FEI Tecnai F20 TEM/STEM (200 kV) and processed with ImageJ software. The samples were baked out at 130°C for 5 hours to remove contaminations before STEM-EELS characterization. The STEM images and EELS maps were collected with a fifth-order aberration-corrected NION UltraSTEM (100 kV) with sub-angstrom resolution. Atomic-scale STEM images were processed with Richard-Lucy deconvolution by three iterations. The Co, Mn, and Fe elemental maps were acquired from their L_3_ edge from EELS spectral image, while the K edge was used for N and O elemental maps. All the elemental maps were processed with principal components analysis with three components and a linear combination of power law for background subtraction in ImageJ software. XPS measurements were carried out using Scienta Omicron ESCA-2SR with monochromatic Al K_α_ x-ray (1486.6 eV) and operating pressure of 1 × 10^−9^ torr. Photoelectrons were collected at a 0° emission angle with a source to analyzer angle of 54.7°. The electron kinetic energy was determined with a hemispherical analyzer, with a pass energy of 200 and 50 eV for survey and high-resolution scans, respectively. The XPS spectra were calibrated with the major adventitious C 1s peak according to (*59*) and analyzed with CasaXPS software assuming a Shirley background.

### Electrochemical measurements

RDE and RRDE measurements were conducted in a conventional three-electrode system with a potentiostat from Pine Instruments. To remove any contamination from noble metals, the three-electrode cell was soaked into aqua regia and rinsed with deionized water three times. A graphite rod was used as the counter electrode, and a Ag/AgCl (saturated KCl) electrode was used as the reference electrode. The potential was calibrated to the RHE scale based on the potential difference (1.0258 V) between Ag/AgCl (saturated KCl) electrode and RHE calculated from the Nernst equation. The working electrode was prepared with catalyst ink by a drop-casting method. The catalyst ink was prepared by dispersing 5 mg of catalyst into 1 ml of Nafion/ethanol (0.25 wt %) dispersion with sonication for 30 min. Ten microliters of ink was drop-casted onto an RDE electrode (glassy carbon disk: 0.1964 cm^2^) to reach a loading of 50 μg cm^−2^. CV measurements were performed in ultrahigh-purity (UHP) Ar-saturated 1 M KOH solution from 0.1 to 1.0 V versus RHE at a sweep rate of 10 mV s^−1^. ORR polarization curves were acquired in UHP O_2_-saturated 1 M KOH solution from 0.3 to 0.95 V versus RHE at a scan rate of 5 mV s^−1^ and rotation speed of 1600 rpm. For ORR selectivity measurements, the same catalyst loading of 50 μg cm^−2^ was used for the RRDE electrode (gassy carbon disk: 0.2475 cm^2^ + Pt ring: 0.1866 cm^2^). The measurement was conducted under similar conditions as the RDE setup. The Pt ring was cleaned with CV scans from 0.05 to 1.2 V versus RHE for 100 cycles at a scan rate of 50 mV s^−1^ before measurements. H_2_O_2_ generated by the ORR process was collected at the Pt ring held at 1.3 V versus RHE with a collection efficiency of 37%. The calculation of H_2_O_2_ yield and electron number can be found in (*13*).

The AEMFC performance with Co_3_N/C cathode was evaluated in an 850e fuel cell test system from Scribner Associates. The preparation details of the catalyst-coated membrane (CCM) can be found in (*22*). Briefly, Co_3_N/C (80 wt %)/ionomer binder (mass ratio of 4:1) and PtRu/C (60 wt %)/ionomer binder (mass ratio of 4:1) dispersed in *n*-propanol were spray-coated onto the two sides of the membrane with an area of 2 × 2 cm^2^ on a hot plate at 80°C. The loading of the anode side was controlled to 0.4 mg_PtRu_ cm^−2^, while the cathode side had a loading of 1.8 mg_Co3N_ cm^−2^. The prepared CCM was soaked in 1 M KOH at 60°C for 12 hours to replace Cl^−^ with OH^−^ and rinsed with deionized water before assembly. The CCM was sandwiched between two pieces of carbon paper (gas diffusion layer) and assembled with bipolar plates and Teflon gaskets at a torque of 6 N·m. The fuel cell testing was carried out at 80°C, with fully humidified O_2_ [500 standard cubic centimeters per minute (sccm)] and H_2_ (500 sccm) with a gas back pressure of 0.2 MPa on both sides. The cell was activated by a five-cycle scan from the open-circuit potential with a step size of 0.2 A and a cutoff voltage of 0.2 V. Stability tests were conducted at a constant cell voltage at 0.76 V.

### Operando XAS measurement and analysis

Co_3_N/C (20 wt %) was dispersed in a Nafion/ethanol (0.25 wt %) solution. Carbon paper was cut into 1 × 5 cm^2^ pieces and used as the catalyst support. The catalyst-ionomer mixture was drop-casted on one end of the carbon paper (1 × 1 cm^2^) using a micropipe and the rest, 1 × 4 cm^2^, served as a nonactive conductor with negligible effects on the catalytic current. The catalyst layer had a low Co_3_N mass loading of 0.2 mg cm^−2^ to ensure that Co_3_N, diluted in the carbon matrix, had a sufficiently low concentration to avoid possible self-absorption in fluorescence mode. As described in our previous cell design (*19*, *60*), the electrochemical cell consists of two pieces of chemically inert polyether ether ketone (PEEK) with an x-ray window in the middle (diameter = 1 mm). One side of the PEEK has a shallow opening to allow the x-ray acquisition at an angle of 45° using a fluorescence detector. Such a cell design allows XAS measurements in both fluorescence and transmission modes. A Teflon U-shaped sealing ring was placed between the two PEEK pieces to adjust the electrolyte thickness to less than 200 μm. On top of the electrochemical cell, a PEEK cap with one gas inlet and one gas outlet was used to bubble N_2_ gas to minimize the effect of trace amounts of O_2_ during the measurement. Inside the electrochemical cell, the catalyst side of the carbon paper was immersed into the electrolyte facing the direction of the incident beam. Like the case of electrochemical measurements, a carbon rod was placed near the working electrode, serving as the counter electrode. The reference electrode [Ag/AgCl (sat. KCl)] was placed (via a salt bridge) at the bottom of the cell to minimize the iR drop during electrochemical testing. All three electrodes were connected to a potentiostat (Biologic SP-200) during operando x-ray data acquisition. Co K-edge XANES spectra were acquired under fluorescence mode at the PIPOX beamline of the Cornell High Energy Synchrotron Source (CHESS). XAS spectra were acquired from 7550 to 8300 eV with a beam spot size of 2 × 1 mm^2^ and averaged from 12 spectra using quadruple fluorescence detectors with three continuous measurements to enhance the S/N ratio. XANES spectra were calibrated based on the characteristic absorption edge of Co metal foils at 7709.0 eV and analyzed using the Athena and Artemis software packages (*61*). Fourier-transformed EXAFS spectra were plotted by applying a Hanning window from 3 to 12 Å^−1^ with *k*^3^-weighting and no phase correction. The EXAFS spectrum of Co_3_N/C catalysts was fitted with the standard crystal structure of Co_3_N (PDF # 04-021-6263).

## Supplementary Materials

### This PDF file includes:

Figs. S1 to S25 Tables S1 to S5 Equation 1 References
