# Supplementary material for: Nonprecious transition metal nitrides as efficient oxygen reduction electrocatalysts for alkaline fuel cells
Source: Sci Adv. 2022 Feb 2;8(5):eabj1584. doi: 10.1126/sciadv.abj1584 (PMC8809680; doi:10.1126/sciadv.abj1584)
Supplement: Supplementary file 1 — Figs. S1 to S25 Tables S1 to S5 Equation 1 References [file sciadv.abj1584_sm.pdf]

Science Advances
AAAS

Supplementary Materials for

Nonprecious transition metal nitrides as efficient oxygen reduction electrocatalysts for alkaline fuel cells

Rui Zeng, Yao Yang, Xinran Feng, Huiqi Li, Lauryn M. Gibbs,
Francis J. DiSalvo, Héctor D. Abruña*

*Corresponding author. Email: hda1@cornell.edu

Published 2 February 2022, *Sci. Adv.* **8**, eabj1584 (2022)
DOI: 10.1126/sciadv.abj1584

This PDF file includes:

Figs. S1 to S25
Tables S1 to S5
Equation 1
References

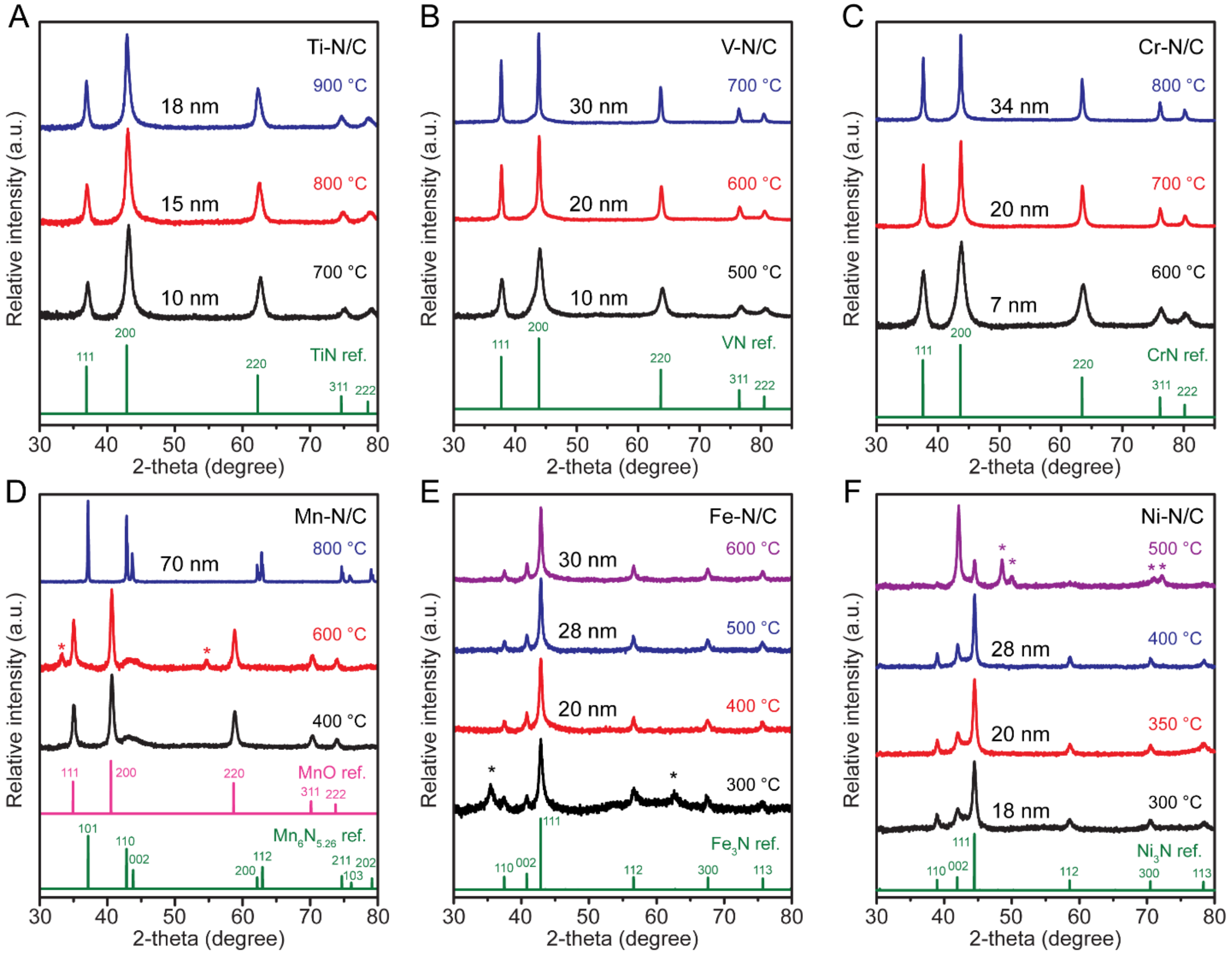

Fig. S1. XRD patterns of 3d metal nitrides prepared at different temperatures. (A) TiN/C synthesized at 700, 800 and 900 ºC and TiN standard reference spectra (C); (B) VN/C synthesized at 500, 600 and 700 ºC and standard VN reference patterns (PCD# 1637900); (C) CrN/C synthesized at 600, 700 and 800 ºC and CrN reference spectra (PCD# 532561); (D) MnN/C synthesized at 400, 600 and 800 ºC and reference patterns of MnO (PCD# 1826194) and $Mn_6N_{5.26}$ (PCD# 1200164); the asterisks at 600 ºC indicate the presence of other phases except MnO; (E) Fe_3N/C synthesized at 300, 400, 500 and 600 ºC and Fe_3N reference patterns (PCD# 1932781); the asterisks at 300 ºC denote the existence Fe_3O_4, suggesting incomplete conversion; (F) Ni_3N/C synthesized at 300, 350, 400, 500 ºC and Ni_3N reference spectra (PCD# 1110883); the asterisks at 500 ºC indicate formation of other products Ni_xN species; The grain sizes were calculated using the Scherrer equation based on the full-width at half-maximum (FWHM) of the major diffraction peaks.

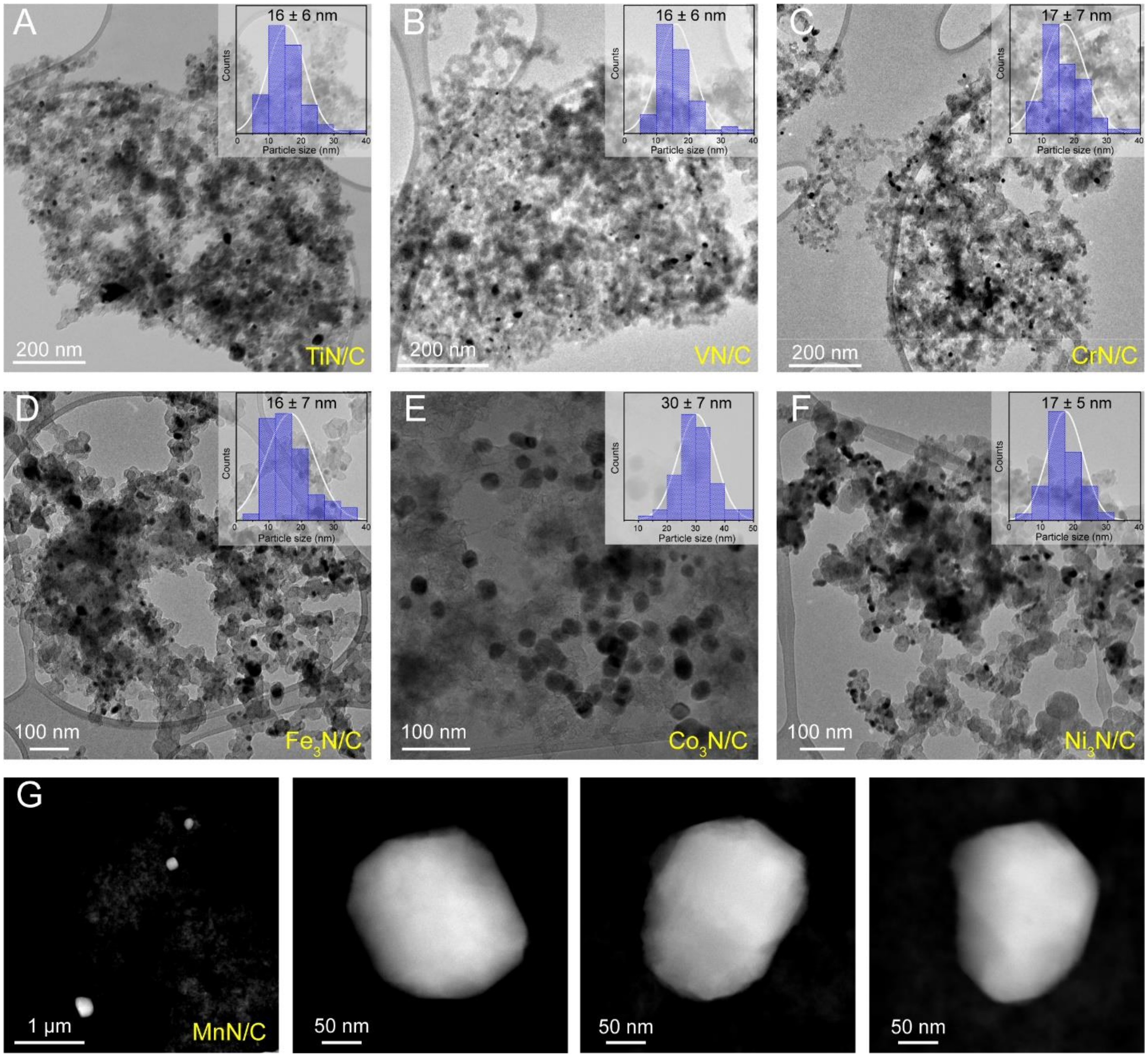

Fig. S2. Morphologies and particle size distributions of as-synthesized metal nitrides. (A) TEM image of TiN/C synthesized 800 ºC, inset shows its particle size distribution (PSD) histogram (16 ± 6 nm); (B) TEM image of VN/C synthesized 600 ºC with PSD histogram (16 ± 6 nm); (C) TEM image of CrN/C synthesized 700 ºC with PSD histogram (17 ± 7 nm); (D) TEM image of Fe_3N/C synthesized 400 ºC with PSD histogram (16 ± 7 nm); (E) TEM image of Co_3N/C prepared at 360 ºC with PSD histogram (30 ± 7 nm); (F) TEM image of Ni_3N/C prepared at 300 ºC with PSD histogram (16 ± 7 nm); (G) STEM images of MnN/C synthesized 800 ºC, particle size was estimated to be ~200 nm.

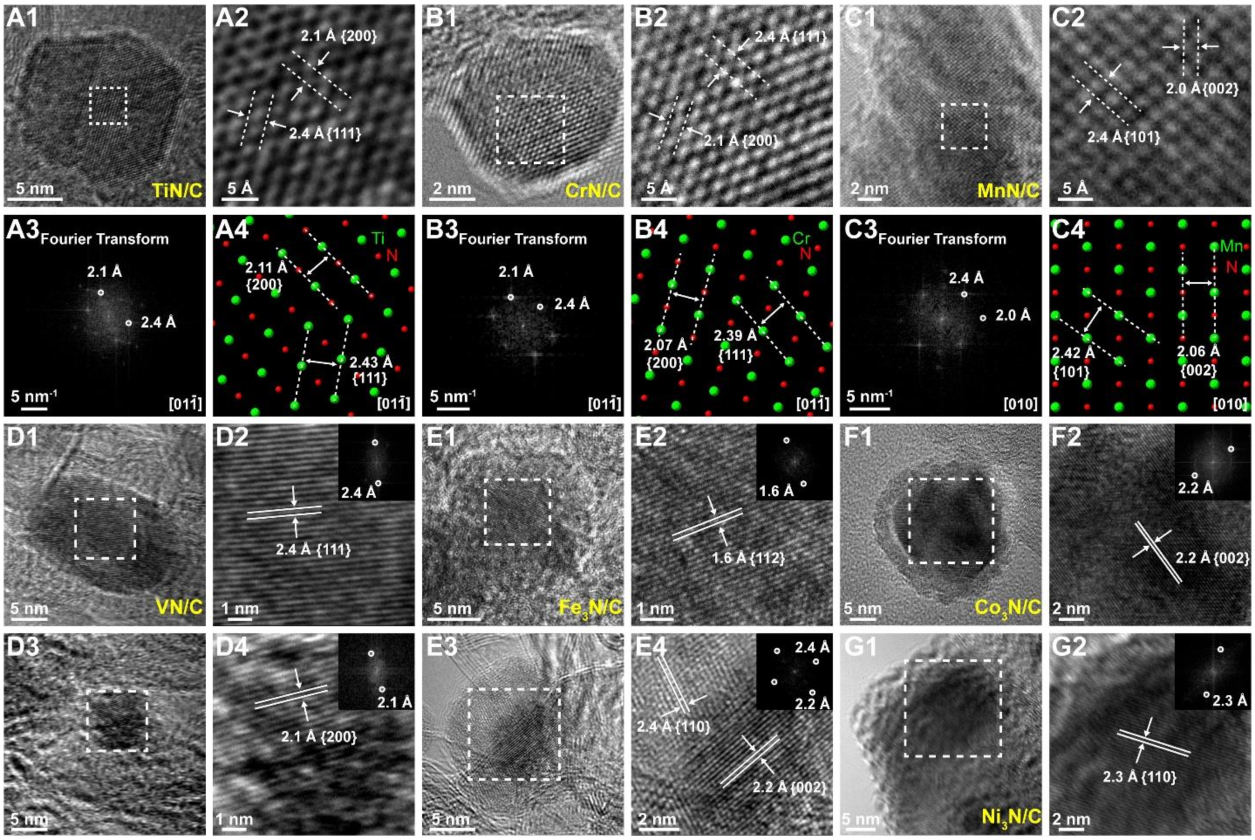

Fig. S3. High-resolution TEM (HRTEM) images of 3d metal nitrides. (A) HRTEM image (A1) and magnified region (A2) of a TiN/C (800 ºC) nanoparticle and its corresponding Fourier transform (A3), consistent with the crystal model viewed from [01$\bar{\mathbf{1}}$] (A4) based on reference TiN; (B) HRTEM image (B1) and zoomed-in observation (B2) of a CrN/C (700 ºC) nanoparticle and its corresponding Fourier transform (B3), consistent with the crystal model viewed from [01$\bar{\mathbf{1}}$] (B4) based on reference CrN; (C) HRTEM image (C1) and zoomed-in observation (C2) of a MnN/C nanoparticle (800 ºC) and its corresponding Fourier transform (C3), in agreement with the crystal model viewed from [010] (C4) based on reference; (D) HRTEM images (D1, D3) and magnified regions (D2, D4) of VN/C nanoparticles (700 ºC) with lattice spacings of 2.4 Å and 2.1 Å, corresponding to VN {111} and {200} facets; (E) HRTEM images (E1, E3) and zoomed-in observations (E2, E4) of Fe_3N/C nanoparticles (400 ºC) with lattice spacings of 1.6, 2.2 and 2.4 Å, corresponding to Fe_3N {112}, {002} and {110} facets, respectively; (F) HRTEM image (F1) and zoomed-in observation (F2) of Co_3N/C nanoparticles (360 ºC) with lattice spacings of 2.2 Å, corresponding to Co_3N {002}; (G) HRTEM image (G1) and zoomed-in observation (G2) of Ni_3N/C nanoparticles (300 ºC) with lattice spacings of 2.2 Å, corresponding to Ni_3N {002}.

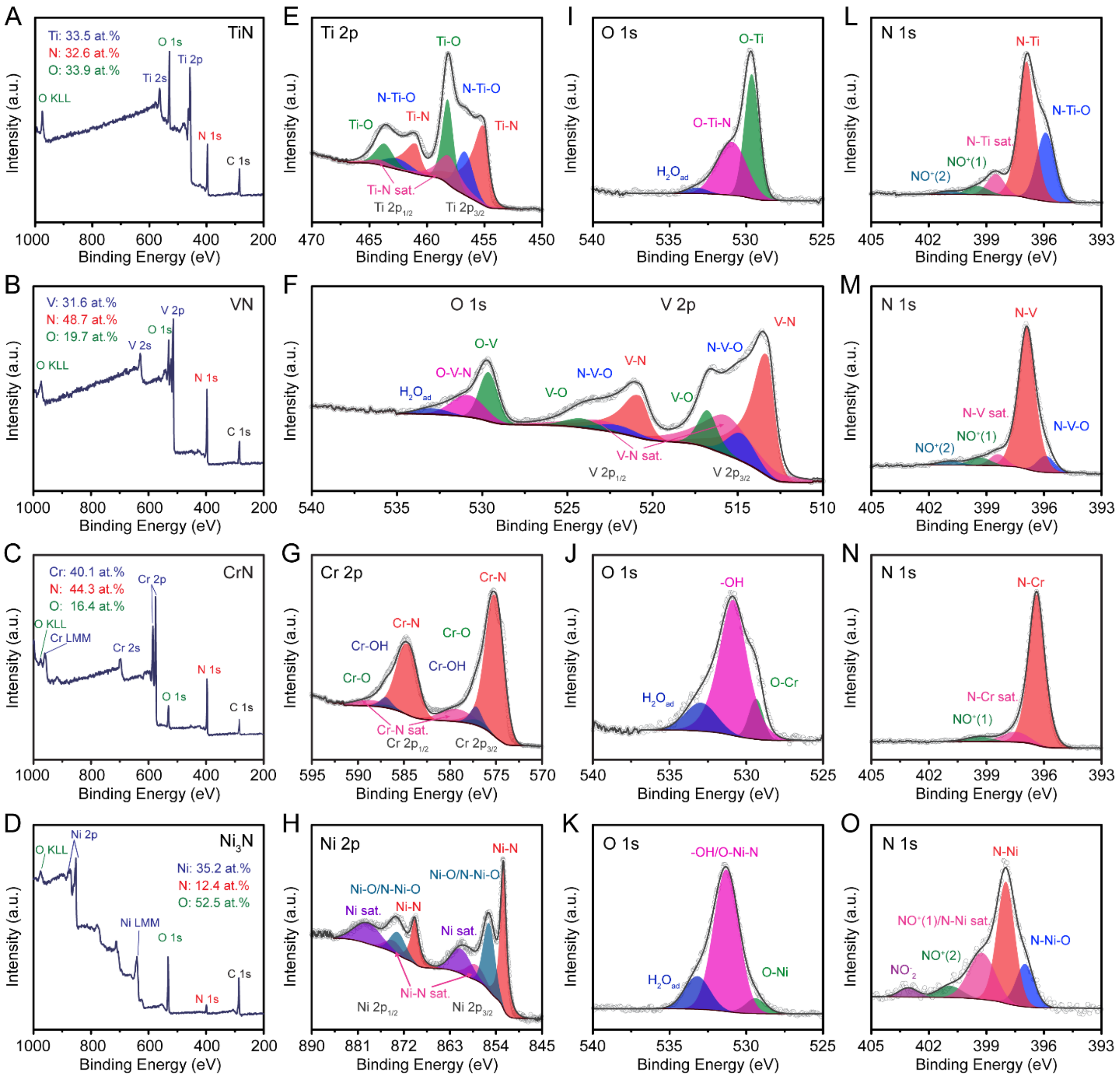

Fig. S4. X-ray photoelectron spectra of as synthesized 3d metal nitrides (TiN, VN, CrN and Ni_3N). (A to D) Survey scans of TiN, VN, CrN and Ni_3N; high-resolution spectra of Ti 2p, O 1s and N 1s signals; The overlapping of V 2p and O 1s spectra led to an underestimate of V and O concentration; (E to H) High-resolution spectra of metal 2p signals; O 1s from VN was analyzed with V 2p spectra; (I to K) High-resolution spectra of O 1s signals; (L to O) High-resolution spectra of N 1s signals. The additional features at higher binding energies in N 1s spectra can be attributed to the oxidized N species (NO^+ or NO_2^-), further providing further evidence for of surface oxidation. The binding energy difference between NO^+(1) and NO^+(2) likely results from NO^+ ions interacting with different oxygen containing species (*62*).

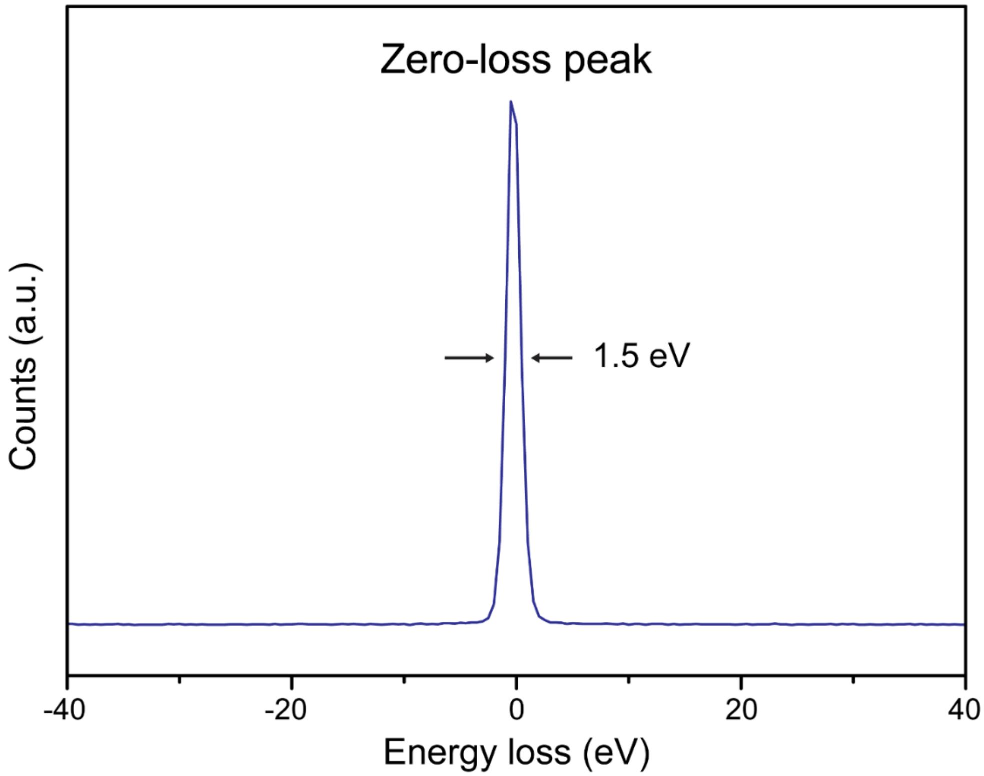

Fig. S5. Zero-loss peak in EELS spectrum with an energy resolution of 1.5 eV. The spectrum was collected with an energy dispersion of 0.5 eV/channel, exposure time of 10 μs and sum frames of 3**.**

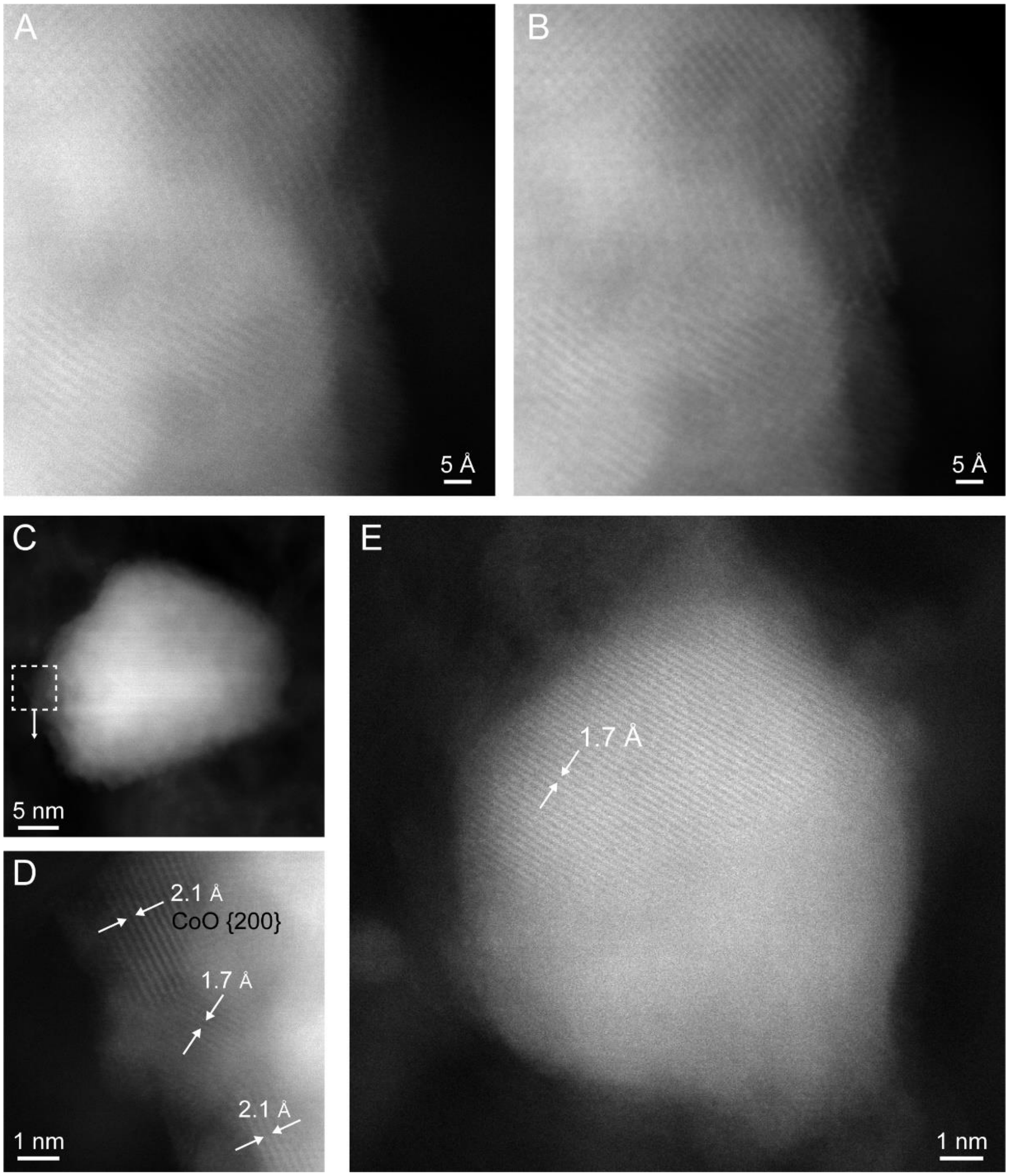

Fig. S6. Atomic-scale STEM images of Co_3N/C. (A to B) Raw STEM image (A) and processed image (B) after Richard-Lucy deconvolution (3 iterations). The deconvolution was processed in ImageJ software assuming an Airy disk (100 kV, α_{max}=28 mrad) convolved with a 0.8 Å Gaussian source image. (C) STEM image of a Co_3N particle. (D) Atomic scale STEM image of particle surface region, acquired from the dashed box region in (C). The 2.1 Å d-spacing value corresponds to CoO{200}, given theoretical d-spacing values: CoO{200} (2.1 Å); Co_3O_4{222} (2.3 Å); {400} (2.0 Å). (E) STEM image of a Co_3N particle with lattice spacings of 1.7 Å. The 1.7 Å possibly arises from either Co_3N{112} (1.6 Å) or Co_3O_4{224} (1.6 Å).

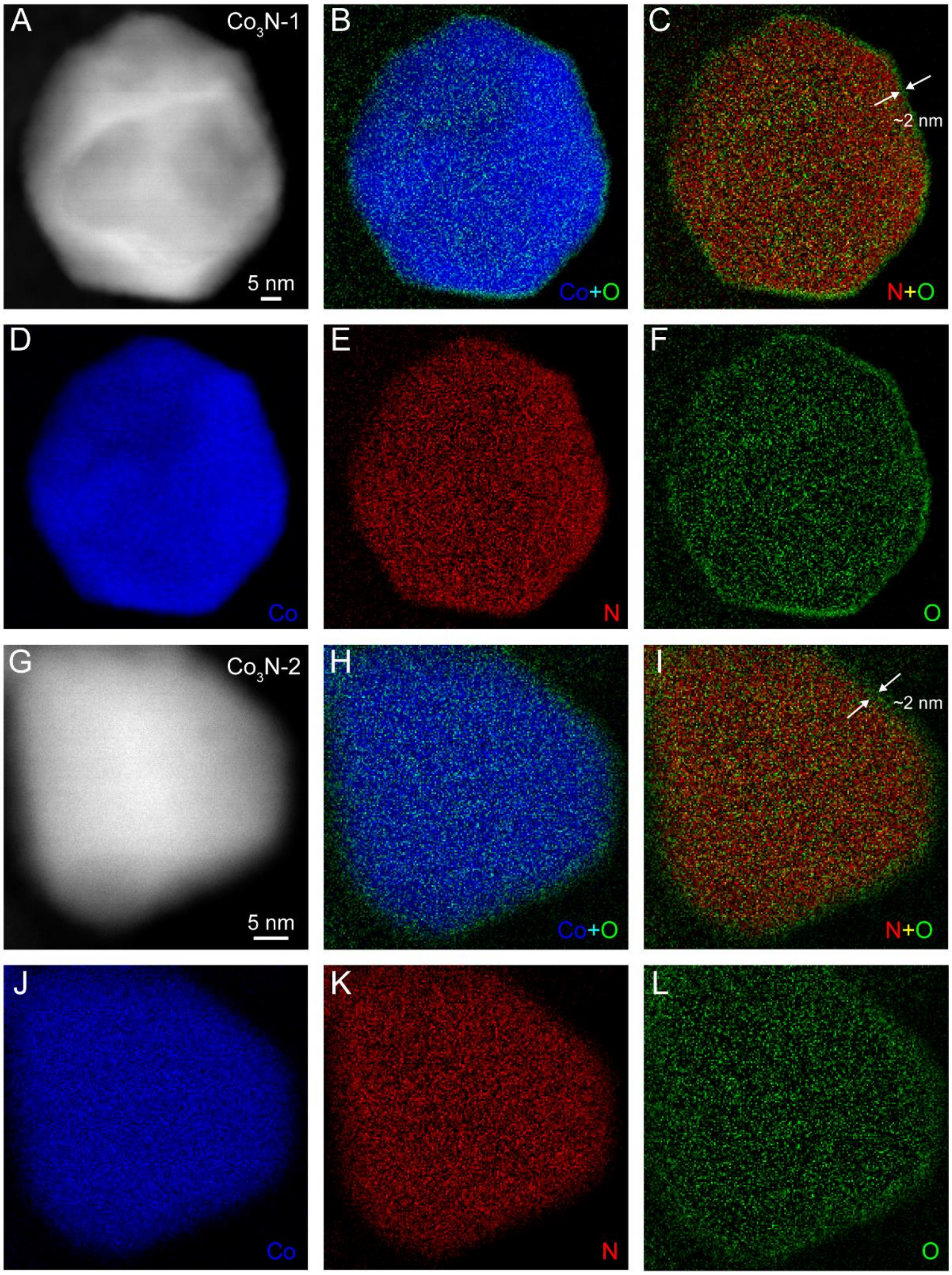

Fig. S7. STEM-EELS analysis of two Co_3N nanoparticles. (A and G) STEM images of Co_3N particles. (B to F and H to L) Corresponding EELS elemental mapping of Co (D, J), N (E, K), O (F, L) and their composite mapping of Co+O (B, H) and N+O (C, I). The elemental mappings clearly manifest the existence of a 2-nm oxide shell over the nitride core; Samples were stored in a N_2 box and transferred to ultra-high vacuum (UHV) chamber with minimum air exposure.

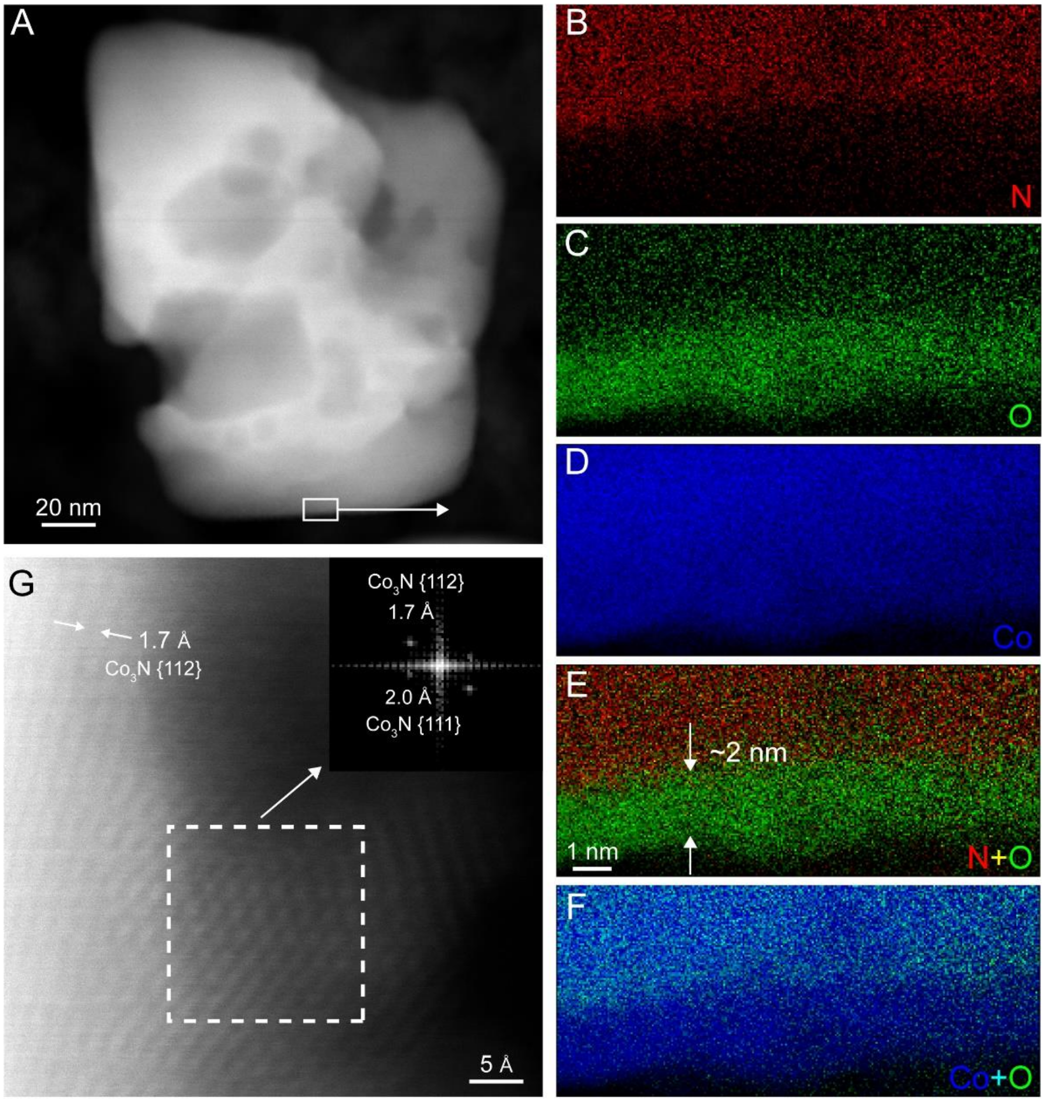

Fig. S8. STEM-EELS analysis of Co_3N particle after exposure to air for 6 months. (A to F) STEM image of Co_3N particle (A) and corresponding elemental mappings of N (B), O (C), Co (D), N+O (E) and Co+O (F). The mappings exhibit a similar 2-nm oxide shell without further oxide layer development, indicating that the core-shell structure is relatively stable in air. (G) Atomic-scale STEM image of a particle region showing the existence of Co_3N with d-spacings of Co_3N\{112\} (1.7 Å) and Co_3N\{111\} (2.0Å) in the inset FFT image, further confirming the overall good stability of Co_3N after being coated by an oxide layer.

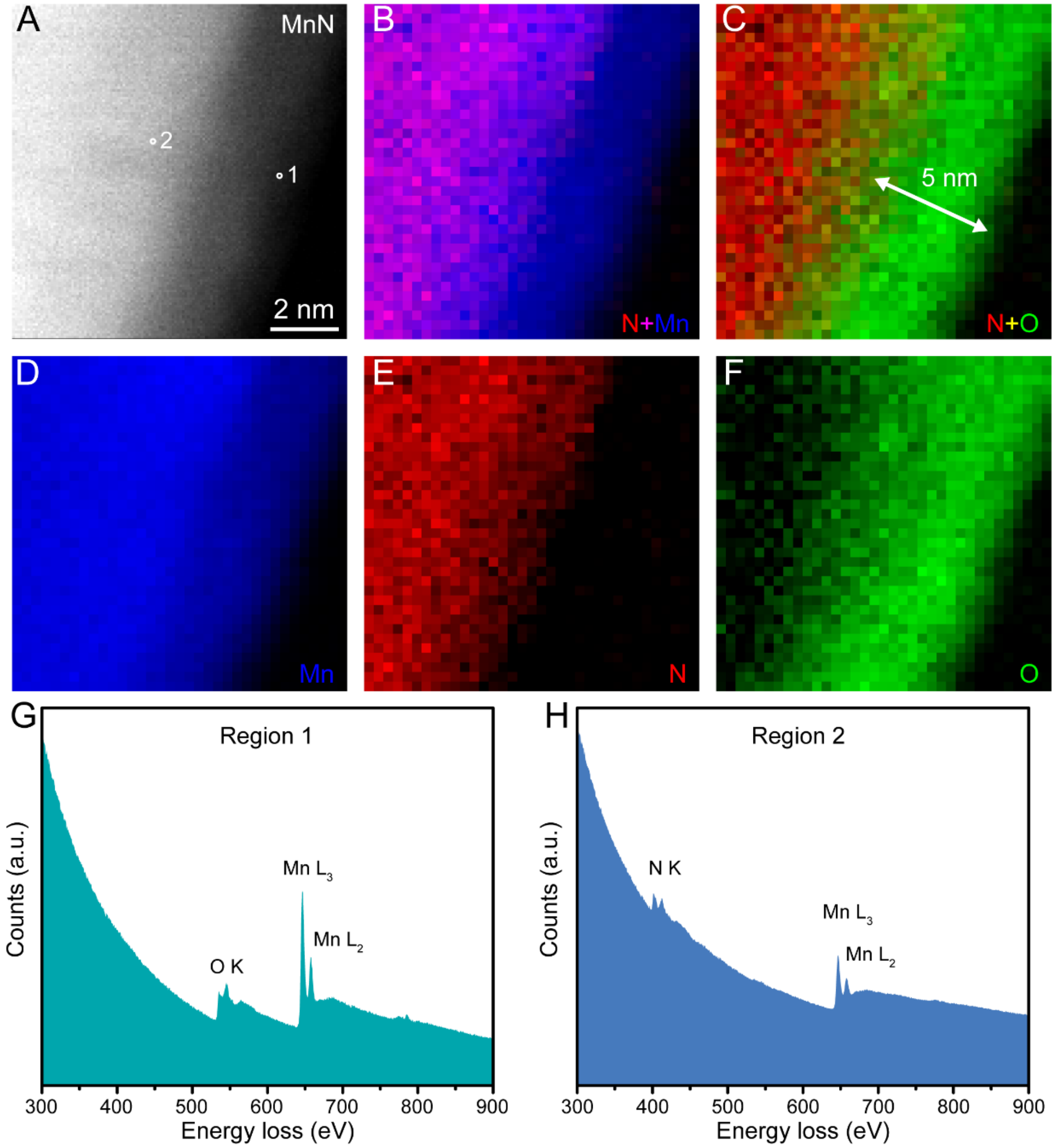

Fig. S9. STEM-EELS analysis of a MnN nanoparticle. (A) STEM image of part of a MnN particle. (B to F) Corresponding EELS elemental mappings of Mn (D), N (E), O (F) and their composite mapping of Mn+N (B) and N+O (C). The elemental mappings clearly provide evidence of the formation of a 5-nm manganese oxide shell over the manganese nitride. (G and H) EELS spectra collected from region 1 (shell) and region 2 (core) in (A). Region 1 displays characteristic O K-edge and Mn L_3 and L_2-edges without presence of N K-edge. Region 2 shows clear features of N K and Mn L-edge. The relatively weaker signals compared to background in region 2 is due to region 2 being much thicker than region 1.

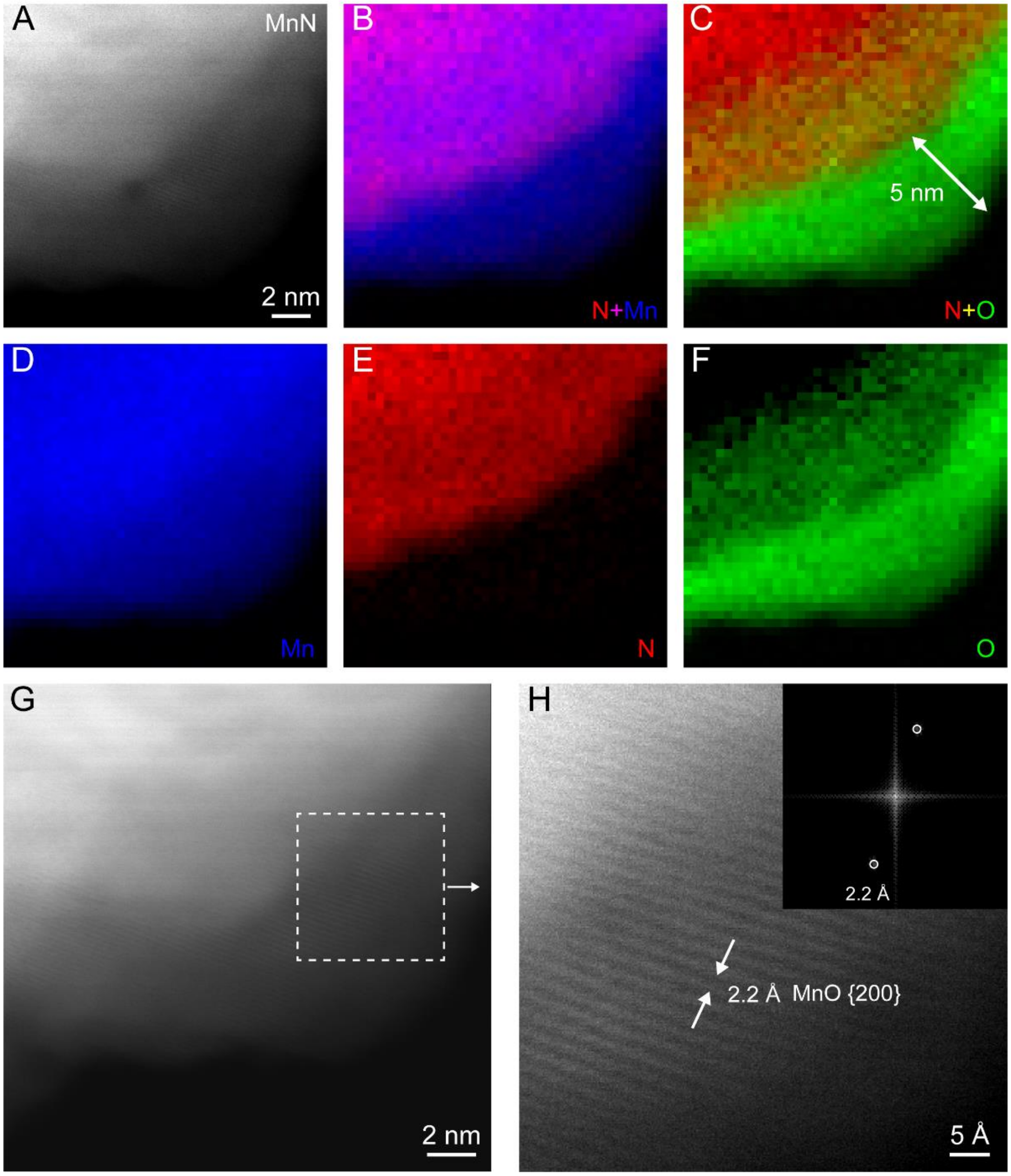

Fig. S10. STEM-EELS analysis of a MnN nanoparticle. (A) STEM image of part of a MnN particle. (B to F) Corresponding EELS elemental mappings of Mn (D), N (E), O (F) and their composite mapping of Mn+N (B) and N+O (C). The elemental mappings present evidence for formation of a 5-nm manganese oxide shell over manganese nitride core. (G) STEM image of a part of MnN particle near the surface. (H) Atomic-scale STEM image acquired from the white dashed box in (G). The image and inset FFT figure show a d-spacing of 2.2 Å, which corresponds to MnO{200}, further suggesting that the particle surface is enriched with manganese oxide.

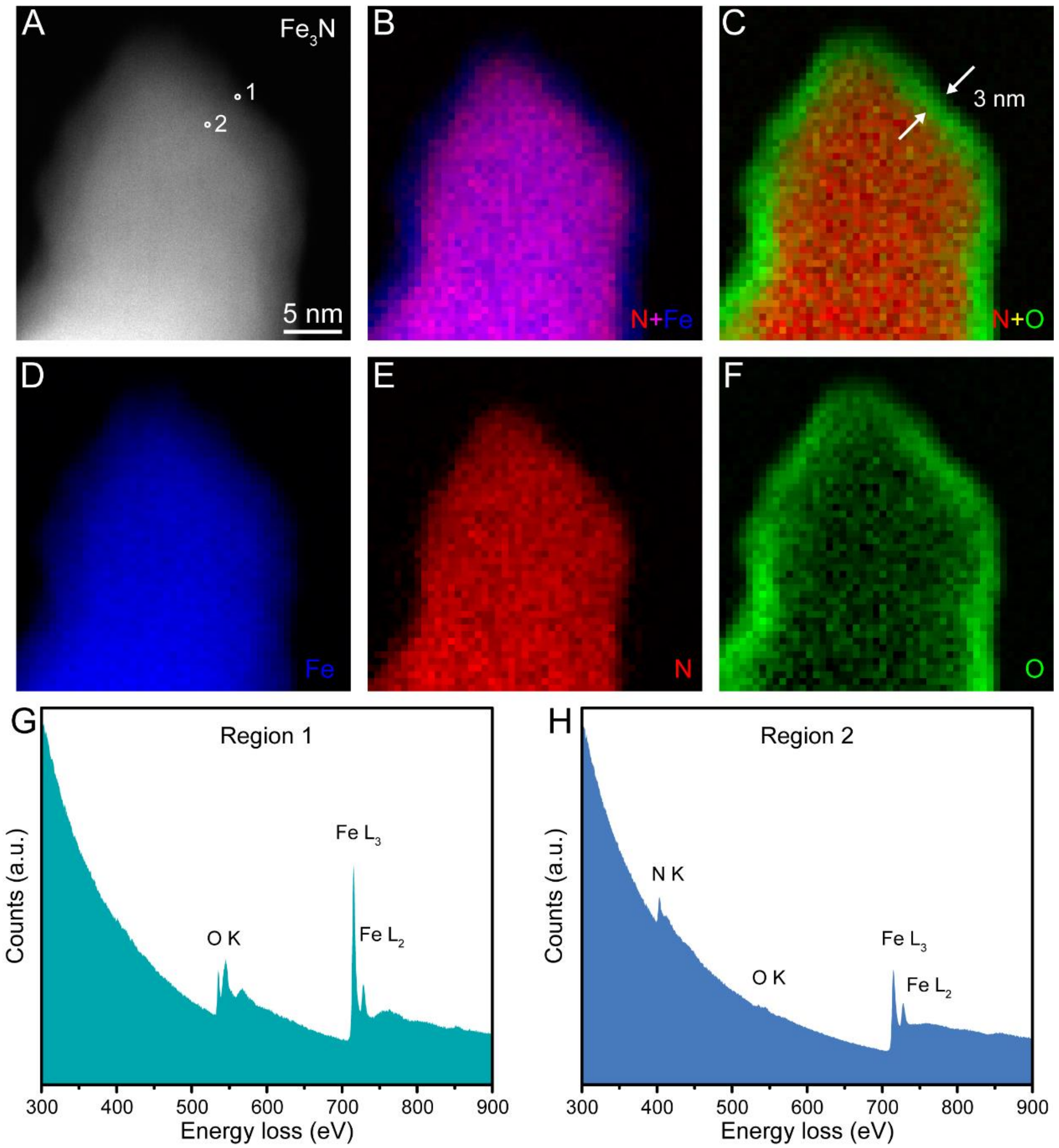

Fig. S11. STEM-EELS analysis of a Fe_3N nanoparticle. (A) STEM image of a Fe_3N particle. (B to F) Corresponding EELS elemental mappings of Fe (D), N (E), O (F) and their composite mapping of Fe+N (B) and N+O (C). The elemental mappings clearly provide evidence for the formation of a 3-nm iron oxide shell over iron nitride core. (G and H) EELS spectra collected from region 1 (shell) and region 2 (core) in (A). Region 1 displays characteristic O K-edge and Fe L_3 and L_2-edges without the presence of the N K-edge. Region 2 shows unambiguous N K and Fe L-edge with negligible O K-edge. The relatively weaker signals compared to background in region 2 arises from region 2 being much thicker than region 1.

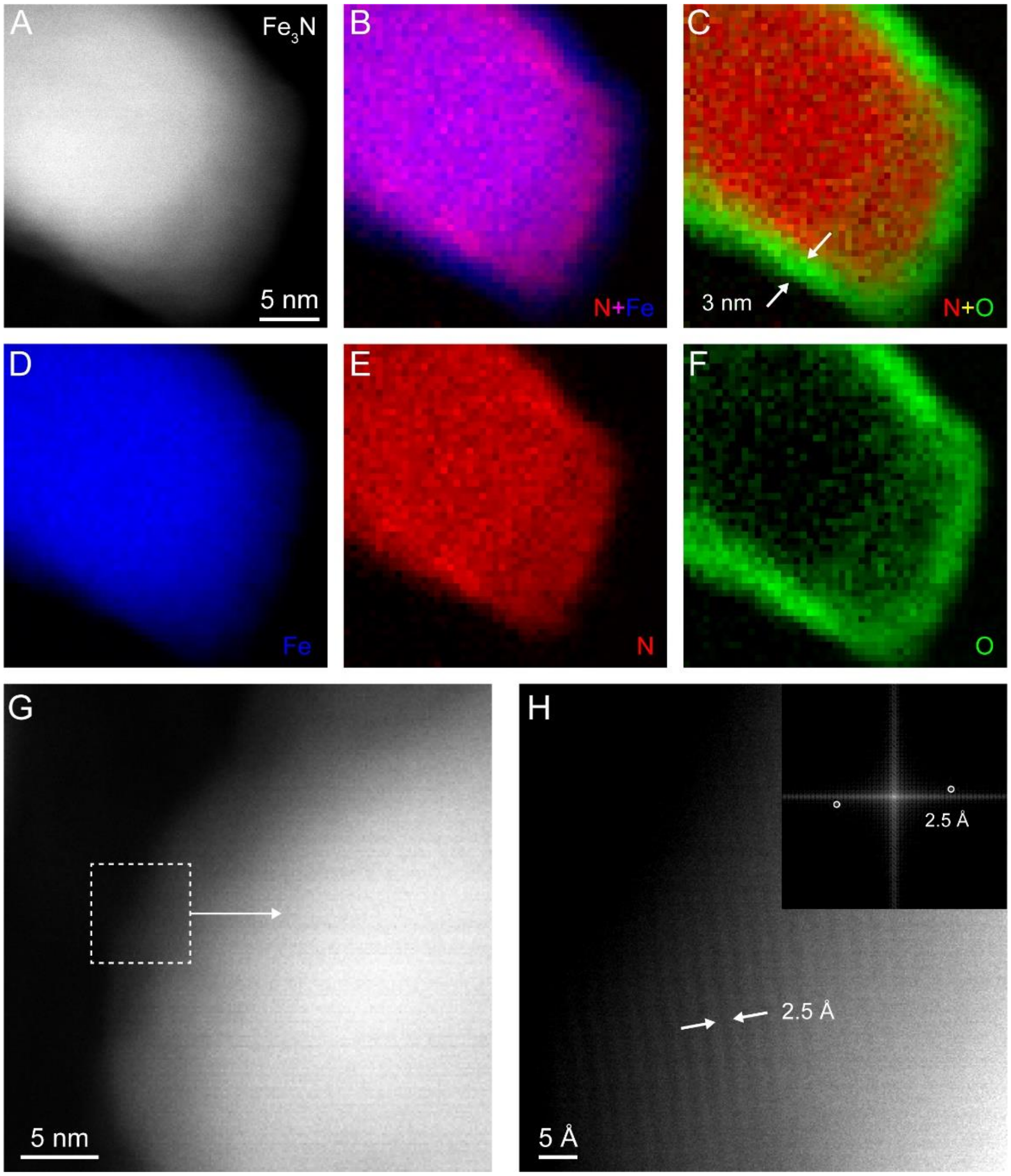

Fig. S12. STEM-EELS analysis of a Fe_3N nanoparticle. (A) STEM image of a Fe_3N particle. (B to F) Corresponding EELS elemental mappings of Fe (D), N (E), O (F) and their composite mapping of Fe+N (B) and N+O (C). The elemental mappings show compelling evidence for the formation of a 3-nm iron oxide shell over iron nitride core. (G) STEM image of a part of Fe_3N particle near the surface. (H) Atomic-scale STEM image acquired from the particle surface as indicated from the white dashed box in (G). The image and inset FFT figure show a d-spacing of 2.5 Å, which may correspond to either FeO{200} or Fe_3O_4{311}, confirming the surface is enriched with iron oxide.

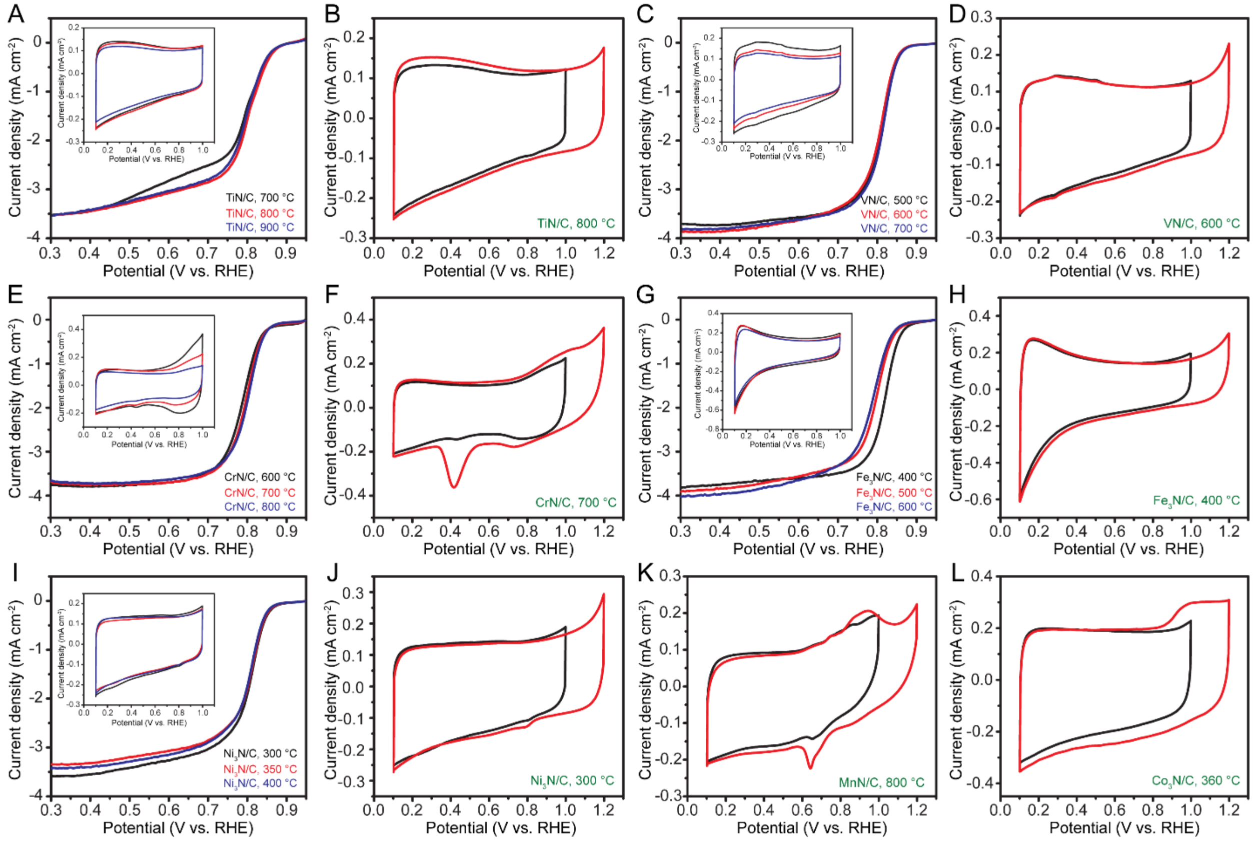

Fig. S13. RDE measurements of 3d metal nitrides synthesized at different temperatures. (A, C, E, G, I) ORR polarization curves and CV profiles (inset) of TiN/C (700, 800 and 900 ºC), VN/C (500, 600 and 700 ºC), CrN/C (600, 700 and 800 ºC), Fe_3N/C (400, 500 and 600 ºC) and Ni_3N/C (300, 350 and 400 ºC), in O_2-saturated 1 M KOH, scan rate 5 mV/s, rotation rate 1,600 rpm. (B, D, F, H & J-L) CV curves with upper limit of 1.0 V and 1.2 V on TiN/C (800 ºC), VN/C (600 ºC), CrN/C (700 ºC), Fe_3N/C (400 ºC), Ni_3N/C (300 ºC), MnN/C (800 ºC) and Co_3N/C (360 ºC), in Ar-saturated 1 M KOH, scan rate 10 mV/s.

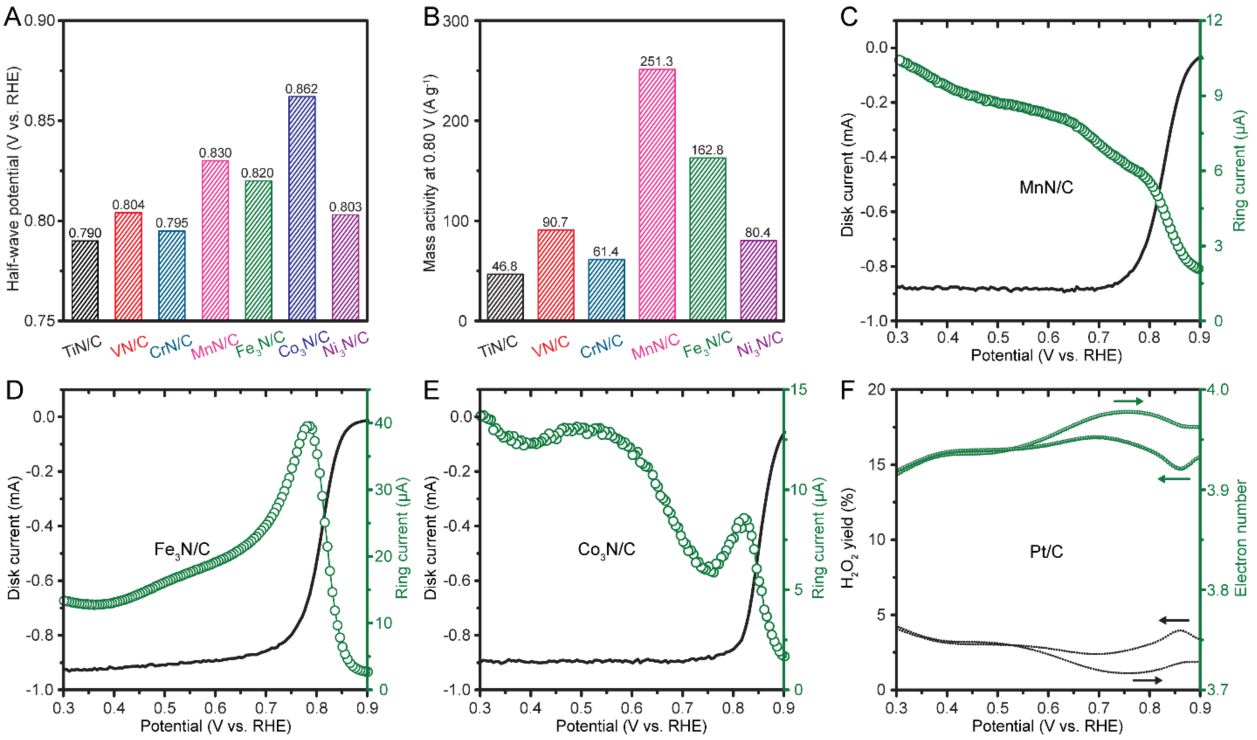

Fig. S14. ORR activity and selectivity of 3d metal nitrides. (A) Half-wave potentials of metal nitrides extracted from ORR polarization curves. (B) Mass activity of metal nitrides determined at a potential of 0.80 V vs. RHE, excluding Co_3N/C. (C to E) RRDE results of MnN/C, Fe_3N/C and Co_3N/C in O_2-saturated 1 M KOH, scan speed 5 mV/s, rotation speed 1,600 rpm. The Pt ring was held at 1.3 V vs. RHE for collection of H_2O_2 generated from the disk. Black solid lines denote disk current while green hollow spots indicate ring current. (F) H_2O_2 yield and electron number of Pt/C calculated from RRDE results with forward and backward scans. The average H_2O_2 yield for Pt/C was estimated to be 3-4% in 1 M KOH solution.

Table S1. Quantitative evaluation of the activity of M_xN/C catalysts. $E_{1/2}$ is the half-wave potential at which the measured current is half of the diffusion-limited current (j_d). E_{onset} is defined as the potential at which the measured current achieves 5% of the diffusion-limited current. MA is the mass normalized kinetic current at 0.85 V (0.80 V) derived from the Koutecký–Levich equation. TS denotes the Tafel slope.

**Catalysts**	**$E_{1/2}$ / V vs. RHE**	**E_{onset} / V vs. RHE**	**j_d / mA cm^{-2}**	**MA / A g^{-1}**	**TS / mV dec^{-1}**
TiN/C	0.790	0.864	3.23	6.4 (46.8)	49
VN/C	0.804	0.861	3.76	6.0 (90.7)	42
CrN/C	0.795	0.856	3.75	4.8 (61.4)	49
MnN/C	0.830	0.893	3.74	27.2 (251.3)	51
Fe_3N/C	0.820	0.874	3.67	12.5 (162.8)	45
Co_3N/C	0.862	0.913	3.75	170.8	37
Ni_3N/C	0.803	0.861	3.41	6.4 (80.4)	44
Pt/C	0.890	0.973	3.74	900	66

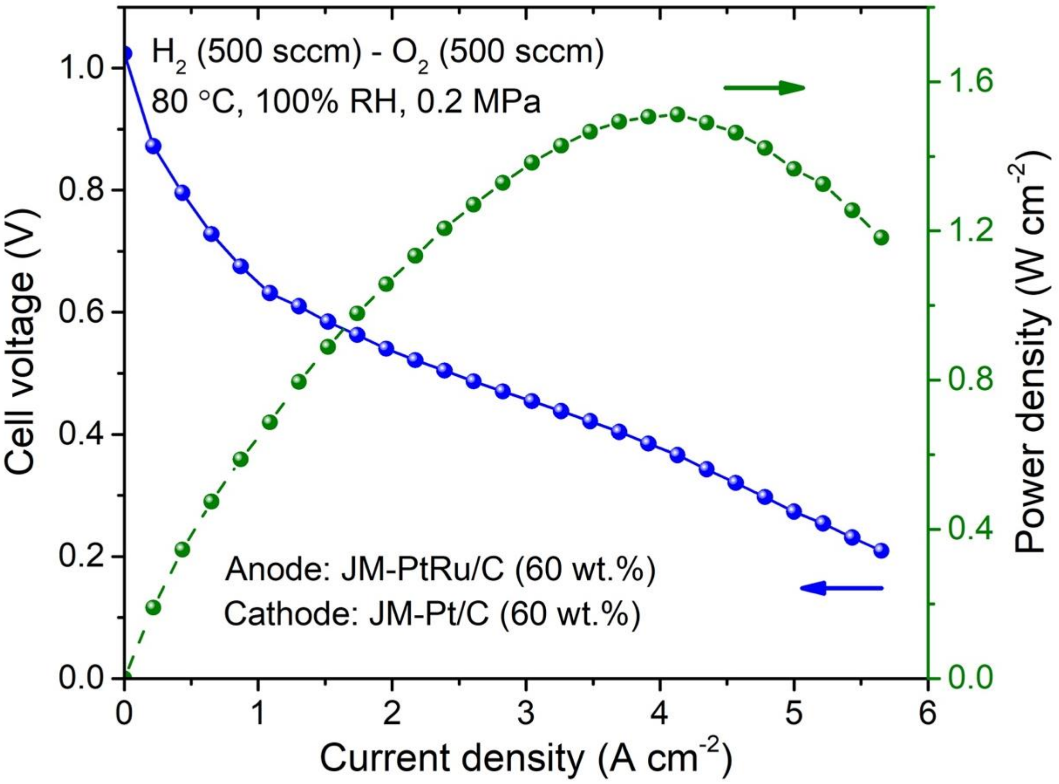

Fig. S15. AEMFC performance with 60 wt.% commercial Pt/C and PtRu/C as cathode and anode catalysts, respectively. The metal loadings were controlled to 0.4 mg cm^{-2} for both catalyst layers. The cell was operated at 80 °C with fully humified H_2 (500 sccm) and O_2 (500 sccm) with a gas back pressure of 0.2 MPa.

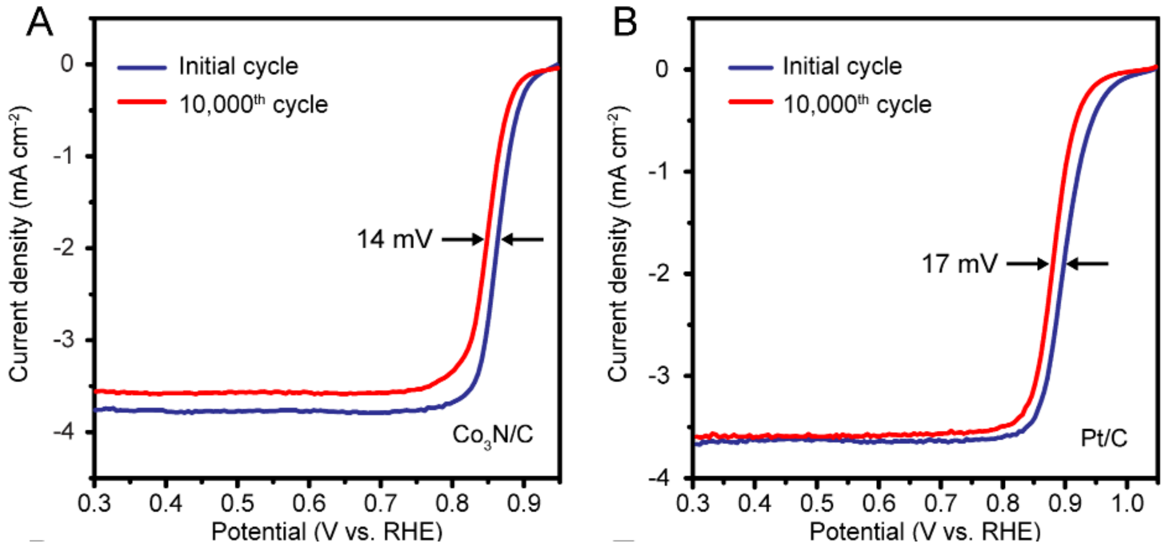

Fig. S16. Comparison of ORR long-term stability between Co_3N/C and Pt/C. ORR polarization profiles of Co_3N/C (A) and Pt/C (B) before and after 10,000 cycles in O_2-saturated 1 M KOH. Scan rate 5 mV/s. The 10,000-cycle stability test (0.60-0.95 V) was carried out in O_2-saturated 1 M KOH, at a sweep rate of 100 mV/s.

Table S2. Cobalt dissolution analysis of Co_3N/C after ADT tests. The dissolved mass was measured by element sensitive inductively coupled plasma mass-spectrometry (ICP-MS)

**Sample**	**Initial mass / µg**	**Dissolved mass / µg**	**Dissolution / %**
ADT (0.6-0.95 V)*	81.4	0.16	0.2
ADT (0.6-1.5 V)**	81.4	0.59	0.7

* The 10,000-cycle ADT test was done over the range of 0.6-0.95 V at a scan rate of 100 mV/s;

**The 10,000-cycle ADT test was done over the range of 0.6-1.5 V at a scan rate of 250 mV/s;

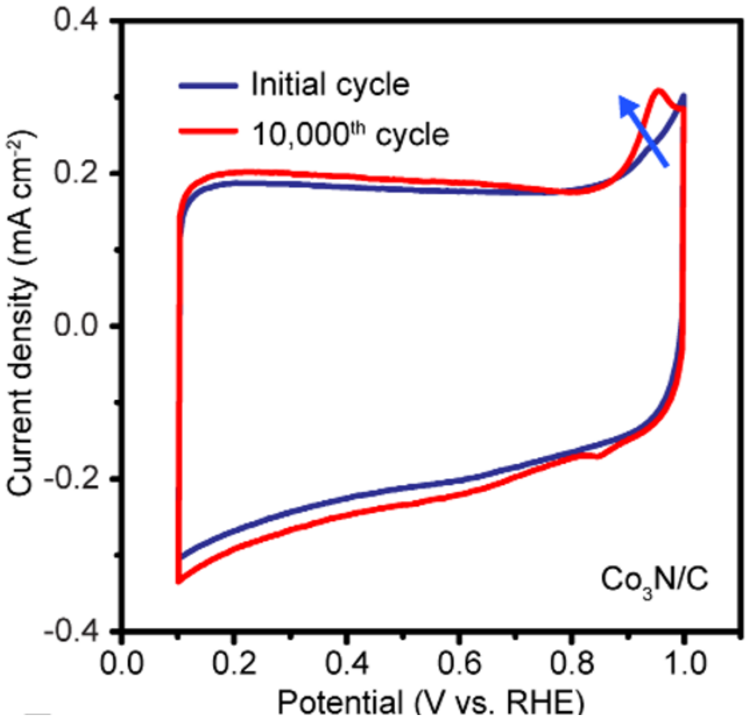

Fig S17. CV profiles of Co_3N/C before and after 10,000 potential cycles. Scan rate of 10 mV/s. The blue arrow indicates the formation of new oxide species after 10,000-cycle stability test.

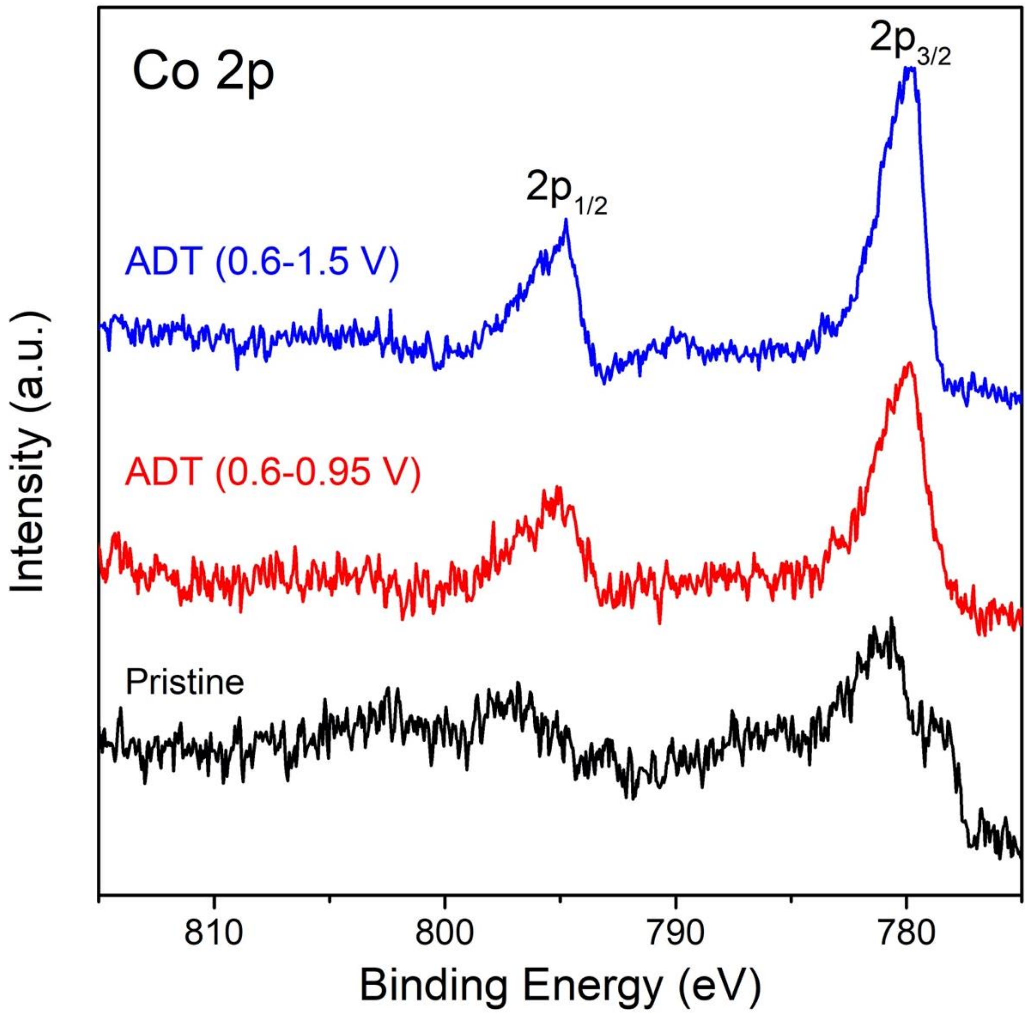

Fig. S18. XPS analysis of Co 2p for Co_3N/C after different ADT tests. The ADT (0.6-0.95 V) and ADT (0.6-1.5 V) samples were tested with 10,000 ADT cycles over the potential range of 0.6-0.95 V and 0.6-1.5 V, respectively.

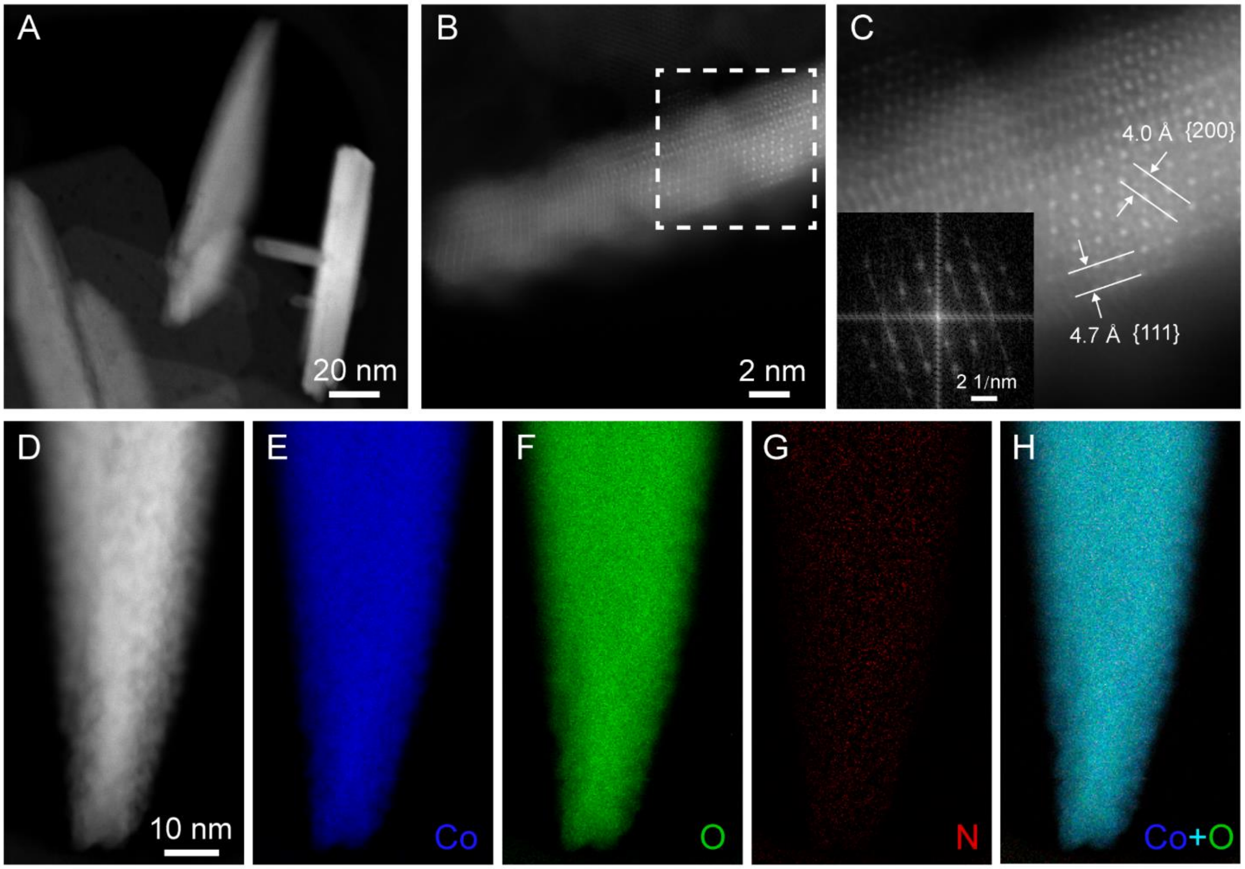

Fig. S19. STEM-EELS analysis of Co_3N/C after 10,000 ADT cycles over the potential range of 0.6-0.95 V. (A-B) STEM images of Co_3N/C after stability testing; (C) Atomic-scale STEM image acquired from the particle indicated in the white dashed box in (B) showing the existence of Co_3O_4 with d-spacings of Co_3O_4{200} (4.0Å) and Co_3O_4{111} (4.7Å); (D to H) STEM image of a rode-like particle (D) and corresponding elemental mapping of Co (E), O (F), N (G) and Co+O (H). The homogenous distribution of Co and O and negligible N signal confirm that the original nitride was completely oxidized to Co_3O_4.

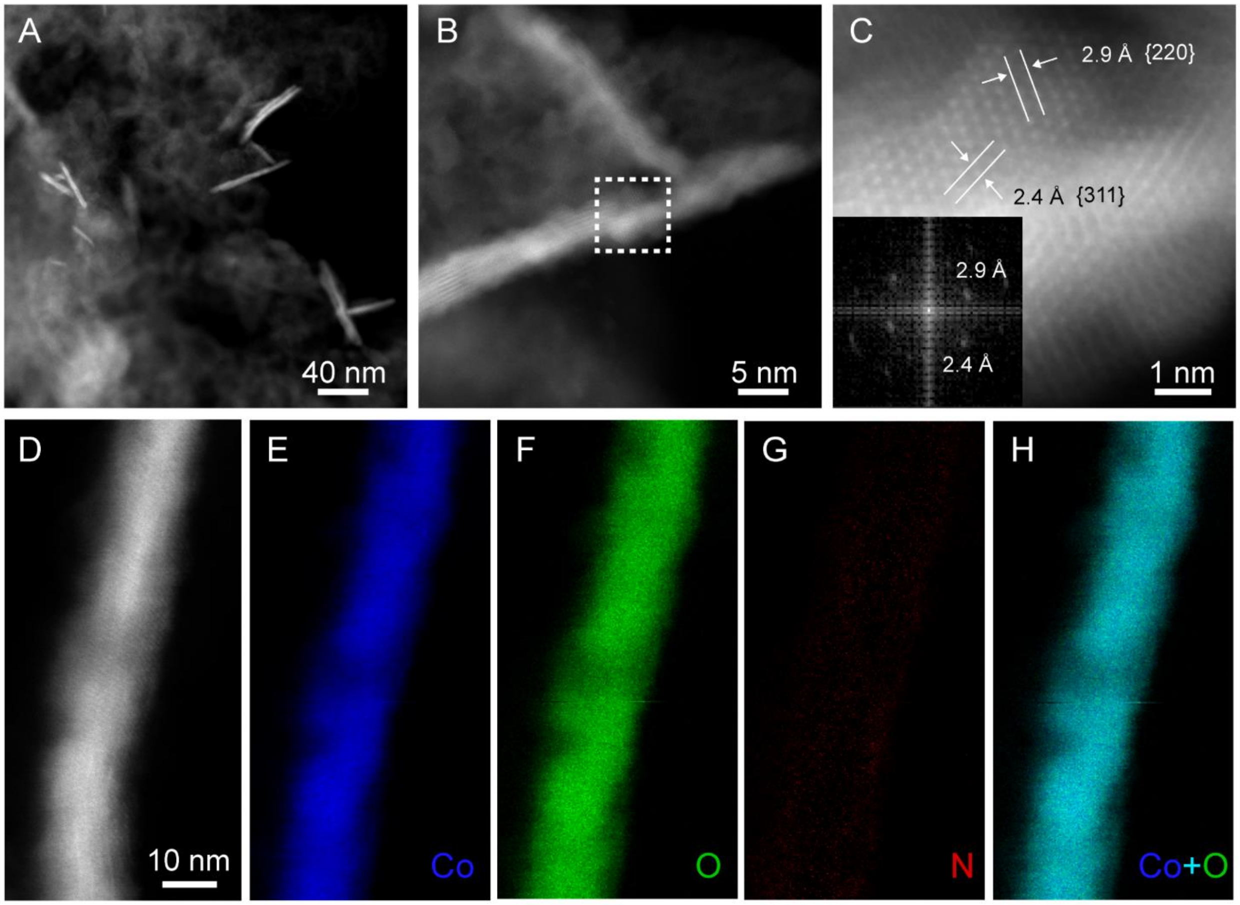

Fig. S20. STEM-EELS analysis of Co_3N/C after 10,000 ADT cycles over the potential range of 0.6-1.5 V. (A-B) STEM images of Co_3N/C after stability testing; (C) Atomic-scale STEM image acquired from the particle indicated in the white dashed box in (B) showing the existence of Co_3O_4 with d-spacings of Co_3O_4{220} (2.9Å) and Co_3O_4{311} (2.4Å); (D to H) STEM image of a rode-like particle (D) and corresponding elemental mapping of Co (E), O (F), N (G) and Co+O (H). The homogenous distribution of Co and O and negligible N signal confirm that the original nitride was completely oxidized to Co_3O_4.

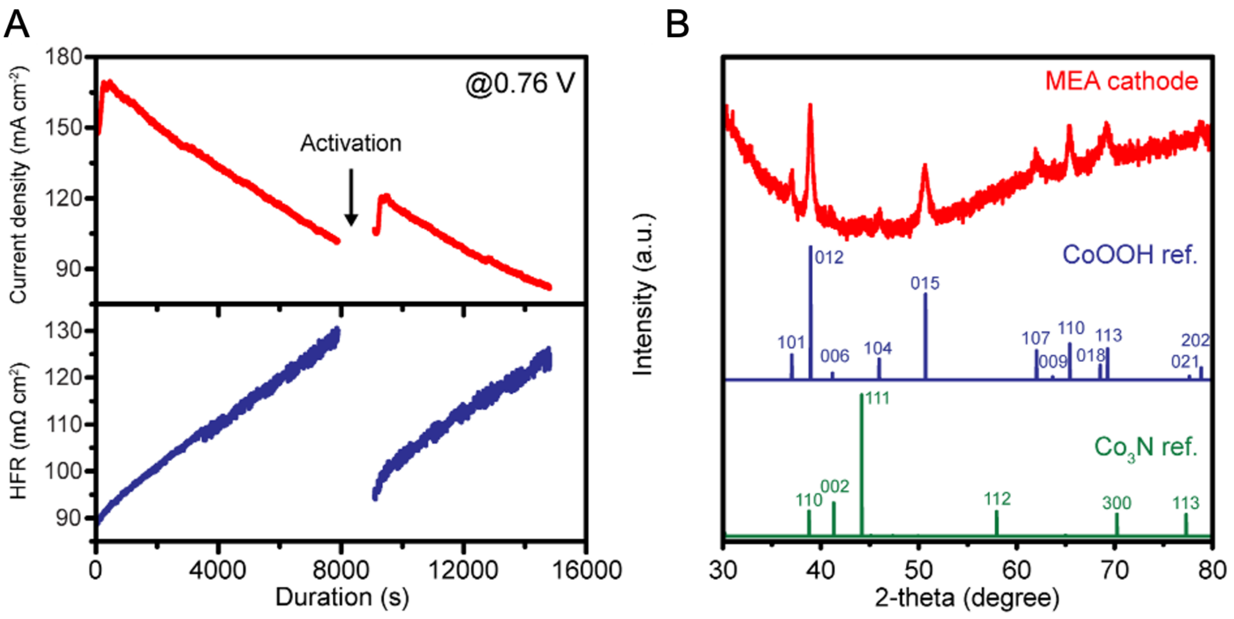

Fig. S21. Stability evaluation of Co_3N/C as cathode catalyst during fuel cell testing. (A) Time response of current density and high-frequency resistance (HFR) at a constant voltage of 0.76 V, tested in a fuel cell with Co_3N/C and PtRu/C as cathode and anode, respectively. HFR was measured at 5,000 Hz. A second activation process was conducted after 8,000 seconds. (B) XRD patterns of MEA cathode after fuel cell test compared with CoOOH (PCD# 1812202) and Co_3N (PCD# 1812202) standard reference spectra. The powder for XRD characterization was obtained by scrapping CCM with a blade. The patterns are in well agreement with CoOOH standard reference, indicating Co_3N underwent degradation into CoOOH phase during fuel cell testing. This may explain the significant decay of the current density.

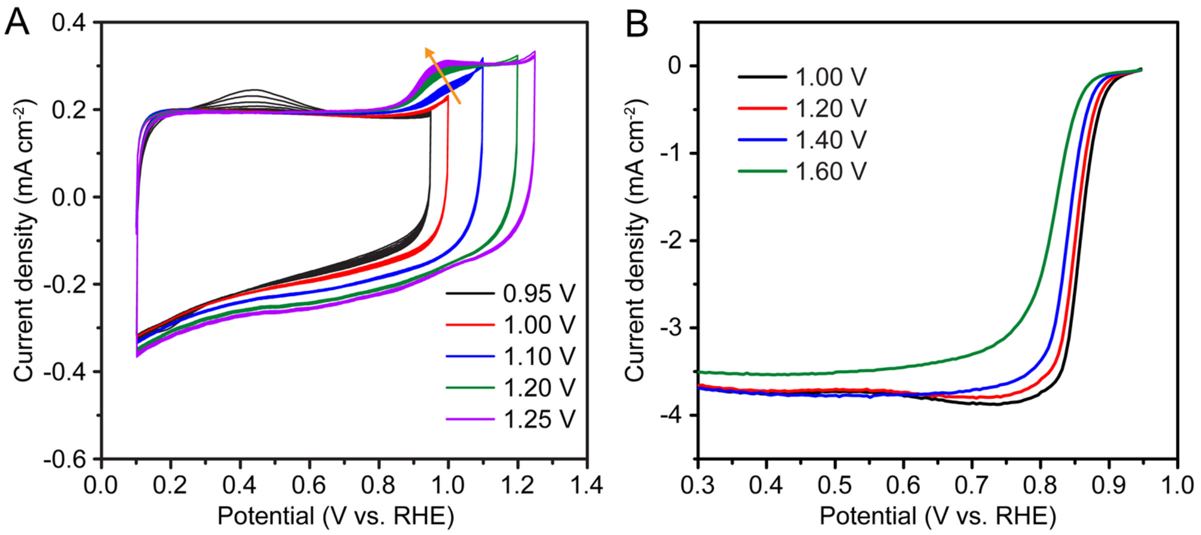

Fig. S22. Effects of CV upper limits on the ORR performance. (A) CV profiles of Co_3N/C at 10 mV/s in Ar-saturated 1M KOH with various upper potential limits from 0.95 V to 1.2 V vs. RHE. A new oxidation peak at ~1.0 V emerged, indicated by the orange arrow, suggesting new specie(s) formed, when the applied potentials were higher than 1.2 V vs. RHE. (B) ORR activity of Co_3N/C before and after 10 CV cycles to 1.2-1.6 V vs. RHE, showing that the treatment at high oxidation potentials decreased the activity.

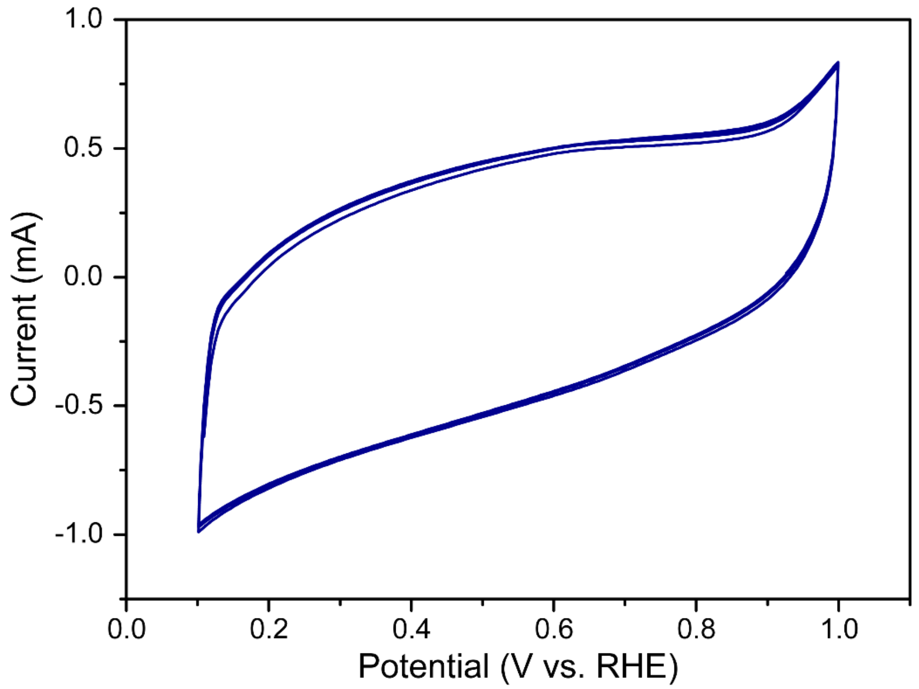

Fig. S23. CV profile of Co_3N/C in *operando* XAS electrochemical cell at 20 mV/s in N_2-saturated 1 M KOH. Co_3N mass loading is 0.2 mg/cm^2 on carbon paper.

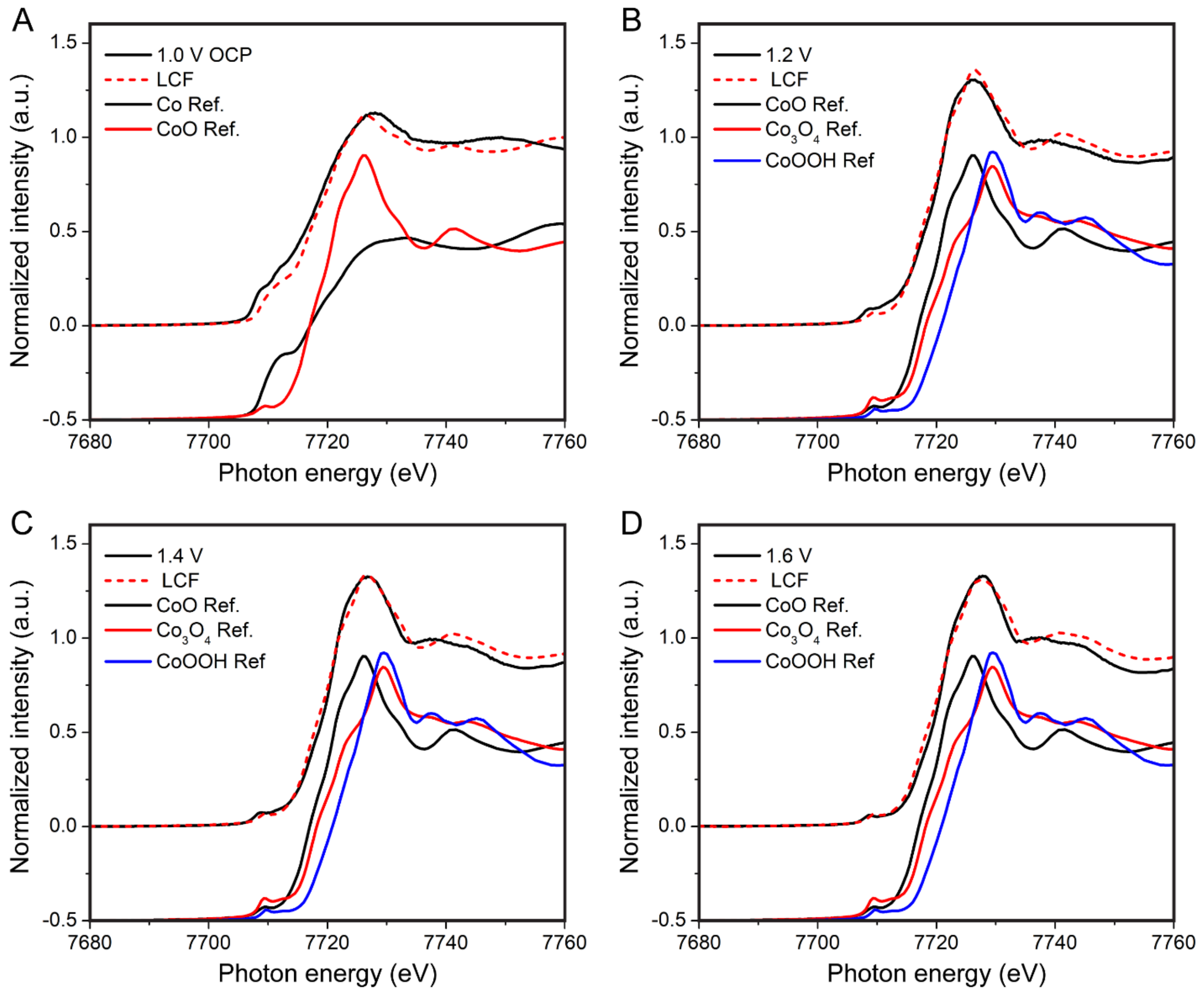

Fig. S24. **Linear combination fitting (LCF) analysis of Co_3N/C at various applied potentials with Co references of metallic Co(0), CoO(II), Co_3O_4(II,III) and CoOOH (III).** The LCF analysis was performed using Athena software and assuming all possible combinations of two, three or four references. Combinations of Co/CoO and CoO/CoOOH were selected for fitting XANES spectra at 1.0 V and 1.2-1.6 V, respectively since they resulted in smallest fitting errors based on the values of reduced χ^2 (chi-square).

Table S3. Calculation of average Co valences at different applied potentials. Reduced χ^2 (chi-square) is a measure of goodness of fit, a value of 0.001 or less suggests a high-quality fitting.

E/ V vs. RHE	Average Co valence	Co at.%	CoO at.%	Co_3O_4 at.%	CoOOH at.%	Reduced χ^2
1.0 V	0.82 ± 0.04	41.3	58.7	0	0	0.00274
1.2 V	2.16 ± 0.02	0	84.3	0	15.7	0.00124
1.4 V	2.23 ± 0.02	0	76.6	0	23.4	0.00137
1.6 V	2.39 ± 0.02	0	60.8	0	39.2	0.00174

$$\chi(k)=\sum_i \frac{\left(N_i S_0^{\ 2}\right)F_i(k)}{kR_i^{\ 2}}\sin\left[2kR_i+\delta_i(k)\right]\mathrm{e}^{-2\sigma_i^{\ 2}k^2}\,\mathrm{e}^{-\frac{2R_i}{\lambda(k)}}$$

Equation S1. EXAFS equation where $F(k)$ and $\delta(k)$ are amplitude and phase shift functions depending on the intrinsic properties of central and scattering atoms. S_0^2 is the amplitude reduction factor often with a value of 0.8~1.0, $\lambda(k)$ is the mean free path for inelastic scattering, N is the coordination number of scattering atoms, σ^2 is the mean square relative displacement (MSRD) to represent disorder (also known as the EXAFS Debye-Waller factor) in coordination environment. Given that the central atom remains the same as Co and the similarity between N and O as scattering atoms, the major factors determining the magnitude of $|\chi(\mathrm{R})|$ in Fig. 5B will be the coordination number, N and level of disorder, σ^2. Given the coordination number of Co-Co remains the same or increases when the structure changes from Co_3N/C to Co oxides (Table S2), a lower magnitude of $|\chi(\mathrm{R})|$ indicates a more disordered structure with larger EXAFS Debye-Waller factor.

Table S4. Standard bond lengths of Co-N, Co-O and Co-Co in Co_3N, CoO, Co_3O_4 and CoOOH. Numbers in parenthesis are coordination numbers, N. Theoretical changes of Co-Co from Co_3N to Co_3O_4 and/or CoOOH are about 0.2 Å, which are consistent with *operando* EXAFS at oxidation potentials. Changes of Co-N in Co_3N to Co-O in Co_3O_4 and/or CoOOH is minimal. Changes of Co-Co or Co-N/O bonds from Co_3N to CoO will be too large compared to experimental observation.

**Standard ref. bond length / Å**	**Co_3N / Å (coordination number, N)**	**CoO / Å (N)**	**Co_3O_4 / Å (N)**	**CoOOH / Å (N)**
Co-N	1.91 (2)	N/A	N/A	N/A
Co-O	N/A	2.13 (6)	1.90 (6) (octahedral)<br>1.96 (4) (tetrahedral)	1.90 (6)
Co-Co	2.61 (2) (nearest)<br>2.65 (4)<br>2.71 (4)<br>2.72 (2)	3.01 (12)	2.85 (6) (nearest)	2.85 (6) (nearest)

Table S5. Radial distance of Co-N/O and Co-Co measured from *operando* EXAFS at oxidizing potentials. Co-Co radial distance showed an upshift from 2.24 to 2.42 by 0.18 Å as potential increased from 1.0 V to 1.6 V. Meanwhile, the Co-N/O distance showed a downshift from 1.52 to 1.40 Å. It should be noted that *operando* EXAFS were processed with no phase correction, thus it is expected to see a phase shift of 0.4-0.5 Å between theoretical bond lengths and experimental radial distance.

E/ V vs. RHE	Co-N/O / Å Based on EXAFS	Co-Co / Å Based on EXAFS
1.0 V	1.52	2.24 (nearest)
1.2 V	1.40	2.1~2.8 (weak, broad)
1.4 V	1.40	2.42
1.6 V	1.40	2.42

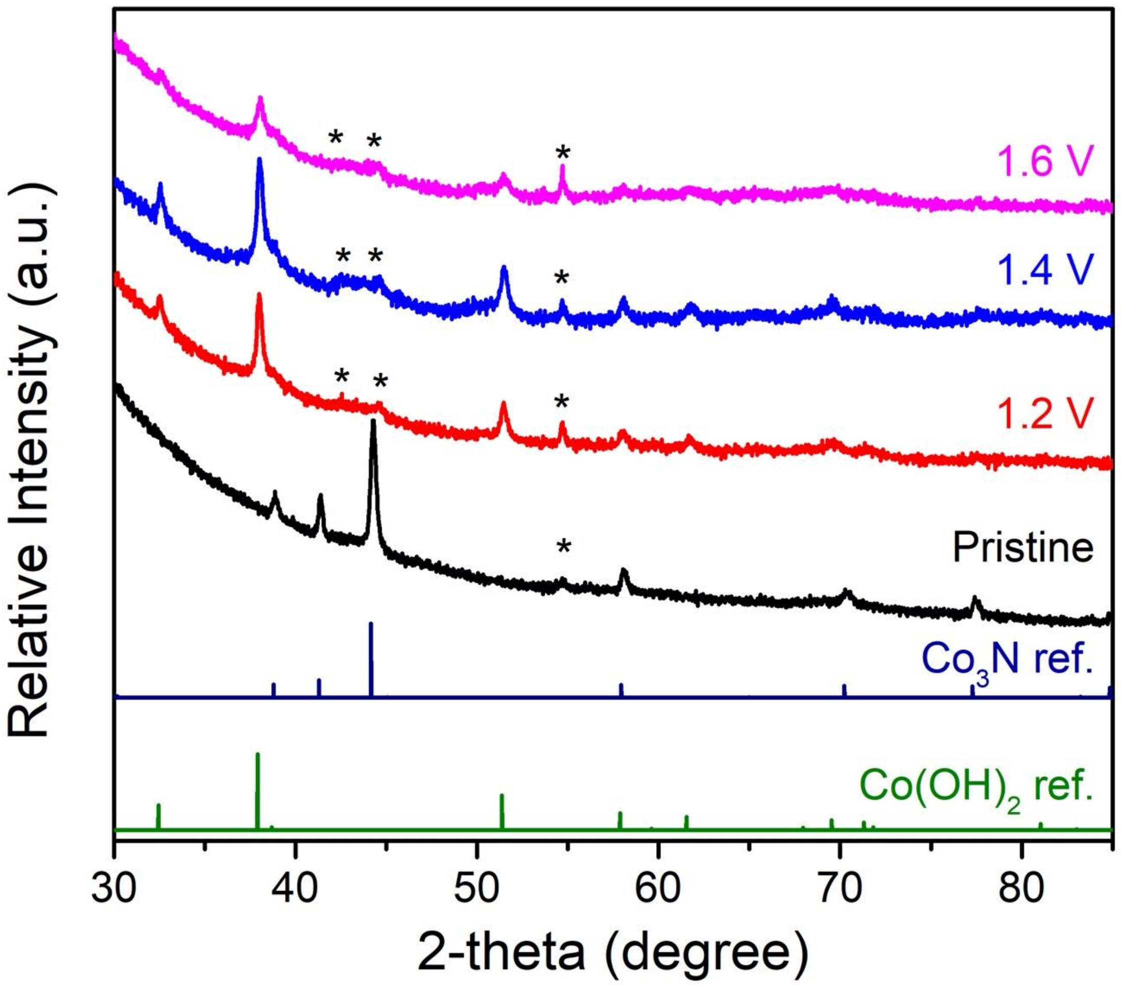

Fig. S25. XRD patterns of Co3N/C before (pristine) and after cycling at 1.2 V, 1.4 V and 1.6 V. The asterisks indicate contributions from the carbon paper.

REFERENCES AND NOTES

1. M. K. Debe, Electrocatalyst approaches and challenges for automotive fuel cells. *Nature* **486**, 43–51 (2012).

2. V. R. Stamenkovic, D. Strmcnik, P. P. Lopes, N. M. Markovic, Energy and fuels from electrochemical interfaces. *Nat. Mater.* **16**, 57–69 (2017).

3. M. Shao, Q. Chang, J.-P. Dodelet, R. Chenitz, Recent advances in electrocatalysts for oxygen reduction reaction. *Chem. Rev.* **116**, 3594–3657 (2016).

4. V. R. Stamenkovic, B. S. Mun, M. Arenz, K. J. J. Mayrhofer, C. A. Lucas, G. Wang, P. N. Ross, N. M. Markovic, Trends in electrocatalysis on extended and nanoscale Pt-bimetallic alloy surfaces. *Nat. Mater.* **6**, 241–247 (2007).

5. D. Wang, H. L. Xin, R. Hovden, H. Wang, Y. Yu, D. A. Muller, F. J. Disalvo, H. D. Abruña, Structurally ordered intermetallic platinum-cobalt core-shell nanoparticles with enhanced activity and stability as oxygen reduction electrocatalysts. *Nat. Mater.* **12**, 81–87 (2013).

6. M. Escudero-Escribano, P. Malacrida, M. H. Hansen, U. G. Vej-Hansen, A. Velázquez-Palenzuela, V. Tripkovic, J. Schiøtz, J. Rossmeisl, I. E. L. Stephens, I. Chorkendorff, Tuning the activity of Pt alloy electrocatalysts by means of the lanthanide contraction. *Science* **352**, 73–76 (2016).

7. Y. Xiong, Y. Yang, H. Joress, E. Padgett, U. Gupta, V. Yarlagadda, D. N. Agyeman-Budu, X. Huang, T. E. Moylan, R. Zeng, A. Kongkanand, F. A. Escobedo, J. D. Brock, F. J. DiSalvo, D. A. Muller, H. D. Abruña, Revealing the atomic ordering of binary intermetallics using in situ heating techniques at multilength scales. *Proc. Natl. Acad. Sci. U.S.A.* **116**, 1974–1983 (2019).

8. M. Li, Z. Zhao, T. Cheng, A. Fortunelli, C. Y. Chen, R. Yu, Q. Zhang, L. Gu, B. V. Merinov, Z. Lin, E. Zhu, T. Yu, Q. Jia, J. Guo, L. Zhang, W. A. Goddard, Y. Huang, X. Duan, Ultrafine jagged platinum nanowires enable ultrahigh mass activity for the oxygen reduction reaction. *Science* **354**, 1414–1419 (2016).

9. X. Tian, X. Zhao, Y. Q. Su, L. Wang, H. Wang, D. Dang, B. Chi, H. Liu, E. J. M. Hensen, X. W. Lou, B. Y. Xia, Engineering bunched Pt-Ni alloy nanocages for efficient oxygen reduction in practical fuel cells. *Science* **366**, 850–856 (2019).

10. Y. Xiong, Y. Yang, F. J. Disalvo, H. D. Abruña, Pt-decorated composition-tunable Pd-Fe@Pd/C core-shell nanoparticles with enhanced electrocatalytic activity toward the oxygen reduction reaction. *J. Am. Chem. Soc.* **140**, 7248–7255 (2018).

11. A. A. Gewirth, J. A. Varnell, A. M. DiAscro, Nonprecious metal catalysts for oxygen reduction in heterogeneous aqueous systems. *Chem. Rev.* **118**, 2313–2339 (2018).

12. H. T. Chung, D. A. Cullen, D. Higgins, B. T. Sneed, E. F. Holby, K. L. More, P. Zelenay, Direct atomic-level insight into the active sites of a high-performance PGM-free ORR catalyst. *Science* **357**, 479–484 (2017).

13. Y. Yang, Y. Xiong, M. E. Holtz, X. Feng, R. Zeng, G. Chen, F. J. DiSalvo, D. A. Muller, H. D. Abruña, Octahedral spinel electrocatalysts for alkaline fuel cells. *Proc. Natl. Acad. Sci. U.S.A.* **116**, 24425–24432 (2019).

14. S. Lu, J. Pan, A. Huang, L. Zhuang, J. Lu, Alkaline polymer electrolyte fuel cells completely free from noble metal catalysts. *Proc. Natl. Acad. Sci. U.S.A.* **105**, 20611–20614 (2008).

15. H. Ren, Y. Wang, Y. Yang, X. Tang, Y. Peng, H. Peng, L. Xiao, J. Lu, H. D. Abruña, L. Zhuang, Fe/N/C nanotubes with atomic Fe sites: A highly active cathode catalyst for alkaline polymer electrolyte fuel cells. *ACS Catal.* **7**, 6485–6492 (2017).

16. S. H. Lee, J. Kim, D. Y. Chung, J. M. Yoo, H. S. Lee, M. J. Kim, B. S. Mun, S. G. Kwon, Y. E. Sung, T. Hyeon, Design principle of Fe-N-C electrocatalysts: How to optimize multimodal porous structures? *J. Am. Chem. Soc.* **141**, 2035–2045 (2019).

17. Y. Liang, Y. Li, H. Wang, J. Zhou, J. Wang, T. Regier, H. Dai, Co_3O_4 nanocrystals on graphene as a synergistic catalyst for oxygen reduction reaction. *Nat. Mater.* **10**, 780–786 (2011).

18. Y. Xiong, Y. Yang, F. J. Disalvo, H. D. Abruña, Metal-organic-framework-derived Co-Fe bimetallic oxygen reduction electrocatalysts for alkaline fuel cells. *J. Am. Chem. Soc.* **141**, 10744–10750 (2019).

19. Y. Yang, Y. Wang, Y. Xiong, X. Huang, L. Shen, R. Huang, H. Wang, J. P. Pastore, S. H. Yu, L. Xiao, J. D. Brock, L. Zhuang, H. D. Abruña, *In situ* x-ray absorption spectroscopy of a synergistic Co-Mn oxide catalyst for the oxygen reduction reaction. *J. Am. Chem. Soc.* **141**, 1463–1466 (2019).

20. J. Suntivich, H. A. Gasteiger, N. Yabuuchi, H. Nakanishi, J. B. Goodenough, Y. Shao-Horn, Design principles for oxygen-reduction activity on perovskite oxide catalysts for fuel cells and metal–air batteries. *Nat. Chem.* **3**, 546–550 (2011).

21. Q. Ji, L. Bi, J. Zhang, H. Cao, X. S. Zhao, The role of oxygen vacancies of ABO3perovskite oxides in the oxygen reduction reaction. *Energ. Environ. Sci.* **13**, 1408–1428 (2020).

22. Y. Yang, H. Peng, Y. Xiong, Q. Li, J. Lu, L. Xiao, F. J. DiSalvo, L. Zhuang, H. D. Abruña, High-loading composition-tolerant Co–Mn spinel oxides with performance beyond 1 W/cm2in alkaline polymer electrolyte fuel cells. *ACS Energy Lett.* **4**, 1251–1257 (2019).

23. Y. Yang, R. Zeng, Y. Xiong, F. J. Disalvo, H. D. Abruña, Cobalt-based nitride-core oxide-shell oxygen reduction electrocatalysts. *J. Am. Chem. Soc.* **141**, 19241–19245 (2019).

24. Y. Zhong, X. H. Xia, F. Shi, J. Y. Zhan, J. P. Tu, H. J. Fan, Transition metal carbides and nitrides in energy storage and conversion. *Adv. Sci.* **3** 1500286 (2015).

25. H. Wang, J. Li, K. Li, Y. Lin, J. Chen, L. Gao, V. Nicolosi, X. Xiao, J.-M. Lee, Transition metal nitrides for electrochemical energy applications. *Chem. Soc. Rev.* **50**, 1354–1390 (2021).

26. Z. Cui, R. G. Burns, F. J. Disalvo, Mesoporous $Ti_{0.5}Nb_{0.5}N$ ternary nitride as a novel noncarbon support for oxygen reduction reaction in acid and alkaline electrolytes. *Chem. Mater.* **25**, 3782–3784 (2013).

27. M. Yang, Z. Cui, F. J. Disalvo, Mesoporous chromium nitride as a high performance non-carbon support for the oxygen reduction reaction. *Phys. Chem. Chem. Phys.* **15**, 7041–7044 (2013).

28. F. Song, W. Li, J. Yang, G. Han, P. Liao, Y. Sun, Interfacing nickel nitride and nickel boosts both electrocatalytic hydrogen evolution and oxidation reactions. *Nat. Commun.* **9**, 4531 (2018).

29. T. Wang, M. Wang, H. Yang, M. Xu, C. Zuo, K. Feng, M. Xie, J. Deng, J. Zhong, W. Zhou, T. Cheng, Y. Li, Weakening hydrogen adsorption on nickelviainterstitial nitrogen doping promotes bifunctional hydrogen electrocatalysis in alkaline solution. *Energ. Environ. Sci.* **12**, 3522–3529 (2019).

30. Y. Yuan, J. Wang, S. Adimi, H. Shen, T. Thomas, R. Ma, J. P. Attfield, M. Yang, Zirconium nitride catalysts surpass platinum for oxygen reduction. *Nat. Mater.* **19**, 282–286 (2020).

31. P. Chen, K. Xu, Z. Fang, Y. Tong, J. Wu, X. Lu, X. Peng, H. Ding, C. Wu, Y. Xie, Metallic Co_4N porous nanowire arrays activated by surface oxidation as electrocatalysts for the oxygen evolution reaction. *Angew. Chem. Int. Ed.* **54**, 14710–14714 (2015).

32. X. Yang, J. Nash, J. Anibal, M. Dunwell, S. Kattel, E. Stavitski, K. Attenkofer, J. G. Chen, Y. Yan, B. Xu, Mechanistic insights into electrochemical nitrogen reduction reaction on vanadium nitride nanoparticles. *J. Am. Chem. Soc.* **140**, 13387–13391 (2018).

33. Y. Zhang, B. Ouyang, J. Xu, G. Jia, S. Chen, R. S. Rawat, H. J. Fan, Rapid synthesis of cobalt nitride nanowires: Highly efficient and low-cost catalysts for oxygen evolution. *Angew. Chem. Int. Ed.* **55**, 8670–8674 (2016).

34. L. Yu, Q. Zhu, S. Song, B. McElhenny, D. Wang, C. Wu, Z. Qin, J. Bao, Y. Yu, S. Chen, Z. Ren, Non-noble metal-nitride based electrocatalysts for high-performance alkaline seawater electrolysis. *Nat. Commun.* **10**, 5106 (2019).

35. J. Luo, X. Tian, J. Zeng, Y. Li, H. Song, S. Liao, Limitations and improvement strategies for early-transition-metal nitrides as competitive catalysts toward the oxygen reduction reaction. *ACS Catal.* **6**, 6165–6174 (2016).

36. A. Miura, C. Rosero-Navarro, Y. Masubuchi, M. Higuchi, S. Kikkawa, K. Tadanaga, Nitrogen-rich manganese oxynitrides with enhanced catalytic activity in the oxygen reduction reaction. *Angew. Chem. Int. Ed.* **55**, 7963–7967 (2016).

37. H. Wu, W. Chen, Copper nitride nanocubes: Size-controlled synthesis and application as cathode catalyst in alkaline fuel cells. *J. Am. Chem. Soc.* **133**, 15236–15239 (2011).

38. K. Khan, A. K. Tareen, M. Aslam, Q. Khan, S. A. Khan, Q. U. Khan, A. S. Saleemi, R. Wang, Y. Zhang, Z. Guo, H. Zhang, Z. Ouyang, Novel two-dimensional carbon–chromium nitride-based composite as an electrocatalyst for oxygen reduction reaction. *Front. Chem.* **7**, 738 (2019).

39. Z. Jin, P. Li, D. Xiao, Enhanced electrocatalytic performance for oxygen reduction via active interfaces of layer-by-layered titanium nitride/titanium carbonitride structures. *Sci. Rep.* **4**, 6712 (2014).

40. H. Yang, H. Al-Brithen, E. Trifan, D. C. Ingram, A. R. Smith, Crystalline phase and orientation control of manganese nitride grown on MgO(001) by molecular beam epitaxy. *J. Appl. Phys.* **91**, 1053–1059 (2002).

41. A. Leineweber, R. Niewa, H. Jacobs, W. Kockelmann, The manganese nitrides η-Mn_3N_2 and θ-$Mn_6N_{(5+x)}$: Nuclear and magnetic structures. *J. Mater. Chem.* **10**, 2827–2834 (2000).

42. K. Suzuki, T. Kaneko, H. Yoshida, Y. Obi, H. Fujimori, H. Morita, Crystal structure and magnetic properties of the compound MnN. *J. Alloys Compd.* **306**, 66–71 (2000).

43. C. Walter, P. W. Menezes, S. Orthmann, J. Schuch, P. Connor, B. Kaiser, M. Lerch, M. Driess, A molecular approach to manganese nitride acting as a high performance electrocatalyst in the oxygen evolution reaction. *Angew. Chem. Int. Ed.* **57**, 698–702 (2018).

44. D. M. Borsa, D. O. Boerma, Phase identification of iron nitrides and iron oxy-nitrides with Mössbauer spectroscopy. *Hyperfine Interact.* **151**, 31–48 (2003).

45. W. Ni, A. Krammer, C. S. Hsu, H. M. Chen, A. Schüler, X. Hu, Ni_3N as an active hydrogen oxidation reaction catalyst in alkaline medium. *Angew. Chem. Int. Ed.* **58**, 7445–7449 (2019).

46. G. Greczynski, L. Hultman, Self-consistent modelling of x-ray photoelectron spectra from air-exposed polycrystalline TiN thin films. *Appl. Surf. Sci.* **387**, 294–300 (2016).

47. M. C. Biesinger, L. W. M. Lau, A. R. Gerson, R. S. C. Smart, Resolving surface chemical states in XPS analysis of first row transition metals, oxides and hydroxides: Sc, Ti, V, Cu and Zn, *Cu and Zn. Appl. Surf. Sci.* **257**, 887–898 (2010).

48. B. P. Payne, M. C. Biesinger, N. S. McIntyre, Use of oxygen/nickel ratios in the XPS characterisation of oxide phases on nickel metal and nickel alloy surfaces. *J. Electron Spectros. Relat. Phenomena* **185**, 159–166 (2012).

49. F. Esaka, K. Furuya, H. Shimada, M. Imamura, N. Matsubayashi, H. Sato, A. Nishijima, A. Kawana, H. Ichimura, T. Kikuchi, Comparison of surface oxidation of titanium nitride and chromium nitride films studied by x-ray absorption and photoelectron spectroscopy. *J. Vac. Sci. Technol. A.* **15**, 2521–2528 (1997).

50. E. Haye, C. Soon Chang, G. Dudek, T. Hauet, J. Ghanbaja, Y. Busby, N. Job, L. Houssiau, J. J. Pireaux, Tuning the magnetism of plasma-synthesized iron nitride nanoparticles: Application in pervaporative membranes. *ACS Appl. Nano Mater.* **2**, 2484–2493 (2019).

51. F. P. Fehlner, *Low-Temperature Oxidation, The Role of Vitreous Oxides* (Wiley, 1986); www.osti.gov/biblio/5328041.

52. M. Luo, Z. Zhao, Y. Zhang, Y. Sun, Y. Xing, F. Lv, Y. Yang, X. Zhang, S. Hwang, Y. Qin, J. Y. Ma, F. Lin, D. Su, G. Lu, S. Guo, PdMo bimetallene for oxygen reduction catalysis. *Nature* **574**, 81–85 (2019).

53. L. Wang, Z. Zeng, W. Gao, T. Maxson, D. Raciti, M. Giroux, X. Pan, C. Wang, J. Greeley, Tunable intrinsic strain in two-dimensional transition metal electrocatalysts. *Science* **363**, 870–874 (2019).

54. R. E. Davis, G. L. Horvath, C. W. Tobias, The solubility and diffusion coefficient of oxygen in potassium hydroxide solutions. *Electrochim. Acta* **12**, 287–297 (1967).

55. K. Chen, K. Liu, P. An, H. Li, Y. Lin, J. Hu, C. Jia, J. Fu, H. Li, H. Liu, Z. Lin, W. Li, J. Li, Y. R. Lu, T. S. Chan, N. Zhang, M. Liu, Iron phthalocyanine with coordination induced electronic localization to boost oxygen reduction reaction. *Nat. Commun.* **11**, 4173 (2020).

56. T. Shinagawa, A. T. Garcia-Esparza, K. Takanabe, Insight on Tafel slopes from a microkinetic analysis of aqueous electrocatalysis for energy conversion. *Sci. Rep.* **5**, 13801 (2015).

57. Y. Yang, Y. Xiong, R. Zeng, X. Lu, M. Krumov, X. Huang, W. Xu, H. Wang, F. J. Disalvo, J. D. Brock, D. A. Muller, H. D. Abrunã, *Operando* methods in electrocatalysis. *ACS Catal.* **11** (2021), 1136–1178.

58. W. Yuan, S. Wang, Y. Ma, Y. Qiu, Y. An, L. Cheng, Interfacial engineering of cobalt nitrides and mesoporous nitrogen-doped carbon: Toward efficient overall water-splitting activity with enhanced charge-transfer efficiency. *ACS Energy Lett.* **5**, 692–700 (2020).

59. G. Greczynski, L. Hultman, X-ray photoelectron spectroscopy: Towards reliable binding energy referencing. *Prog. Mater. Sci.* **107**, 100591 (2020).

60. Y. Xiong, Y. Yang, X. Feng, F. J. Disalvo, H. D. Abruña, A strategy for increasing the efficiency of the oxygen reduction reaction in Mn-doped cobalt ferrites. *J. Am. Chem. Soc.* **141**, 4412–4421 (2019).

61. B. Ravel, M. Newville, ATHENA, ARTEMIS, HEPHAESTUS: Data analysis for x-ray absorption spectroscopy using IFEFFIT*J. Synchrotron Radiat.* **12**, 537–541 (2005).

62. J. Torres, C. C. Perry, S. J. Bransfield, D. H. Fairbrother, Low-temperature oxidation of nitrided iron surfaces. *J. Phys. Chem. B* **107**, 5558–5567 (2003).
